# Disrupted Copper Homeostasis and Impaired Retinal Development Caused by *slc6a4a* Deficiency in Zebrafish

**DOI:** 10.3390/ani16132036

**Published:** 2026-07-02

**Authors:** Hameed Ullah Baloch, Yuan-Yuan Jing, Jia-Hao Shi, Han-Fei Wang, You Wu, Jing-Xia Liu

**Affiliations:** 1 Key Laboratory of Freshwater Animal Breeding, College of Fisheries, Ministry of Agriculture, Huazhong Agricultural University, Wuhan 430070, China; hameedbaloch062@yahoo.com (H.U.B.); jyy1772996858@163.com (Y.-Y.J.); jiahaos1999@163.com (J.-H.S.); hansen123419@163.com (H.-F.W.); 2 Yangtze River Fisheries Research Institute, Chinese Academy of Fishery Sciences, Wuhan 430223, China; wuyou@yfi.ac.cn

**Keywords:** *slc6a4a*, copper homeostasis, Atp7b, retinal development, tetrathiomolybdate

## Abstract

Serotonin transporter Slc6a4a functions as a transporter in serotonin reuptake and is tightly linked with serotonergic regulation and stress responses. However, few studies have investigated its role in copper homeostasis and organogenesis in an in vivo vertebrate model. In this study, we demonstrate that *slc6a4a* deficiency (*slc6a4a^−/−^*) leads to copper accumulation, retinal developmental defects, and locomotor dysfunction in zebrafish specifically. Mechanistically, *slc6a4a* deficiency is associated with reduced *atp7b* and copper accumulation, which lead to reactive oxygen species (ROS) and endoplasmic reticulum (ER) stress, and results in Caspase-3-mediated apoptosis and retinal degeneration. Specifically, tetrathiomolybdate (TTM), a pharmacological copper chelator, partially reduces ER stress and restores retinal defects. Additionally, ectopic expression of full-length *atp7b* mRNA partially restores retinal defects. These findings identify serotonin transporter Slc6a4a as a novel regulator in copper homeostasis and retinal development via the regulation of Atp7b in an in vivo vertebrate model. This study supports a mechanistic link between *slc6a4a* deficiency, copper overload, and retinal defects and highlights copper chelation as an alternative therapeutic strategy in individuals with *Slc6a4* deficiency.

## Simple Summary

Mutations in SLC6A4 (solute carrier family 6 member 4) are closely linked to neurotransmission, stress, and anxiety-like behaviors, and SLC6A4 has recently been identified as a modulator of copper homeostasis in in vitro HeLa cells. However, the relationship among Slc6a4a deficiency, copper homeostasis, and neurogenesis has not been studied in an in vivo vertebrate model such as zebrafish. This study reveals that *slc6a4a* deficiency (*slc6a4a^−/−^*) in zebrafish leads to abnormal locomotion, copper accumulation, and retinal abnormalities specifically. *Slc6a4a* deficiency is associated with reduced *atp7b* expression and copper accumulation, which increases reactive oxygen species (ROS) production and endoplasmic reticulum (ER) stress, resulting in retinal degeneration. Our study also shows that full-length *atp7b* mRNA partially reduces ER stress and that tetrathiomolybdate (TTM) (a copper chelator) partially rescues retinal degeneration.

## 1. Introduction

Serotonin (5-hydroxytryptamine; 5-HT) is a monoamine signaling molecule that regulates multiple physiological and behavioral processes in vertebrates, including digestion, heart rate, reproduction, vascular resistance, respiration, stress response, and other biological processes [1,2,3]. Solute carrier family 6 member 4 (Slc6a4) encodes a serotonin (5-HT) transporter (SERT) [4], which is essential for neurotransmitter signaling and consequent neuronal activity. It uptakes 5-HT from the synapse, which helps to terminate neurotransmitter signals and limit 5-HT-induced neuronal activity, allowing 5-HT to be recycled and stored [5]. The SERT (Slc6a4) protein and transcripts are found in numerous brain regions, such as the retina, but are most prominent in the midbrain raphe nuclei [6]. However, the roles of *Slc6a4a* in neurogenesis remain incompletely defined.

Slc6a4 is highly conserved in vertebrates, particularly in teleost fish, and has been comprehensively described in zebrafish [7,8], which is a frequently used model organism in developmental and neurobiological research. Zebrafish (*Danio rerio*) have a complex serotonergic system featuring all major genes for 5-HT synthesis, metabolism, and signaling, with two genes encoding serotonin transporter homologs, *slc6a4a* and *slc6a4b* [9]. Recently, SLC6A4 has been identified as a novel modulator of copper homeostasis in human cells in vitro [10]. Copper (Cu) is a vital trace element that plays essential roles during organismal development and growth [11], and Cu homeostasis is pivotal for neurogenesis, such as retinal cell development [12,13,14]. Cu overload is known to be toxic to cells, resulting in protein degradation and cell apoptosis [15]. In this study, we investigated the relationship among Slc6a4a, Cu homeostasis, and retinal cell development in an in vivo vertebrate model, zebrafish. We generated a *slc6a4a* mutant (*slc6a4a^−/−^*, with a 10 bp deletion in exon 1) zebrafish to investigate its role during organogenesis. We hypothesized that the loss of *slc6a4a* would disrupt copper homeostasis, leading to developmental defects in neurogenesis.

## 2. Materials and Methods

### 2.1. Ethics Statement

All animal experiments were performed in compliance with the “Guidelines for Experimental Animals” approved by the Institutional Animal Care and Use Ethics Committee of Huazhong Agricultural University (HZAUFI-2021-0018).

### 2.2. Similarity Comparison for Zebrafish Slc6a4a

Multiple sequence alignment was carried out using MEGA 12 software (https://www.megasoftware.net/releases/MEGA_12.0.14_win64_setup.exe (accessed on 28 June 2026)) to determine the evolutionary similarity of zebrafish Slc6a4a and Slc6a4b to human SLC6A4. A phylogenetic tree was created to show orthologous relationships among species based on SLC6A4 protein sequences using the online tool Phylogeny.fr IQ-TREE 2.4.0 (https://phylogeny.fr/tools/iqtree) (accessed on 28 June 2026).

### 2.3. Generation of slc6a4a Mutants

Zebrafish *slc6a4a* homozygous mutants (*slc6a4a^−/−^*) were generated using CRISPR/Cas9 genome editing technology, with gRNA targeting exon 1 (5′-AGAGAACGGCAGGCTGGTTG-3′, PAM: TGG). A mutant line with a 10 bp deletion in exon 1 was constructed and used in this study. The primers used to genotype heterozygous (*slc6a4a^+/−^*) and homozygous *slc6a4a (slc6a4a^−/−^*) are listed in Supplementary Table S1.

### 2.4. Zebrafish Maintenance and Embryo and Larvae Collection

Adult wild-type (WT) zebrafish (AB strain) and *slc6a4a^−/−^* were maintained in a circulating water system under standard laboratory conditions at 28 ± 0.5 °C with a 14 h light/10 h dark photoperiod. Fish were fed three times daily (9:00 AM, 3:00 PM, 9:00 PM) with commercial Artemia (Tianjin Fengnian Aquatic Products Cultivation Co., Ltd., Tianjin, China). Male and female zebrafish were kept separately before mating and spawning. After spawning naturally, the fertilized embryos were pooled, cleaned, and collected under a dissection microscope (SMZ168, Motic, Xiamen, China). The age of embryos, larvae, and adults was staged according to a standard process in the China Zebrafish Resource Center (http://www.zfish.cn/), using hours post-fertilization (hpf), days post-fertilization (dpf), or months, respectively.

### 2.5. Inductively Coupled Plasma Mass Spectrometry (ICP-MS)

The total copper content in zebrafish embryos was measured by inductively coupled plasma mass spectrometry (ICP-MS). The copper (Cu) level in each sample was analyzed as previously described [16,17]. The samples were digested using highly purified HNO_3_, following with a microwave digestion program at 140 °C and 180 °C. The samples were subsequently evaporated to remove acid and resolved in 2% electronic-grade HNO_3_ to a final volume of 5 mL. After vigorous vortexing, each digested solution was centrifuged for 10 min at 10,000 rpm, and the supernatant was used for ICP-MS analysis. Copper ion standards prepared in 2% HNO_3_ at 0, 0.1, 1, 10, 25, 50, and 100 μg/L were used for calibration. Rhodium at 20 μg/L was used as an internal standard to correct signal drift and matrix effects. Copper levels were blank-corrected and normalized to sample mass using the equation: ω = (C_sample − C_blank) × V × D/(m × 1000), where ω is expressed as mass ratio measured in units μg/g, C represents concentration value measured in units of μg/L, V is the final volume of the digested solution, D is the dilution factor, and m is the sample wet weight (g).

### 2.6. mRNA Synthesis and Microinjection

The full-length coding sequences of zebrafish *atp7b* and *slc6a4a* were amplified and used to synthesize mRNAs in vitro. The synthesized mRNAs were used to test the specificity of phenotypic defects induced by *slc6a4a* deficiency and to test whether *atp7b* mediates the roles of *slc6a4a* in retinal development. In this study, a Message Machine kit (AM1340, Ambion, Waltham, MA, USA) was used to synthesize capped *atp7b* and *slc6a4a* mRNAs in vitro, according to the manufacturer’s instructions. The synthesized mRNA was diluted to 200 ng/μL and injected into fertilized one-cell stage embryos, following a previously described protocol [18]. The embryos and larvae from different groups were collected for the following assays.

### 2.7. Cu Chelator Tetrathiomolybdate (TTM) Treatment

In this study, WT and *slc6a4a^−/−^* embryos and larvae were co-treated with copper chelator tetrathiomolybdate (TTM) [19] to reduce the copper level in embryos and their retinal cells. TTM (Sigma-Aldrich, Cat. No. 323446, St. Louis, MO, USA) was dissolved in DMSO (Biosharp, Beijing, China, Cat. No. BS08) at 20 mM to prepare a stock solution and was diluted in embryo medium to a final concentration of 200 μM as reported previously [20] at the bud stage for treatment in this study. The experimental groups, including WT, *slc6a4a^−/−^*, WT + TTM, WT + DMSO, and *slc6a4a^−/−^* + TTM, were used, and each group was replicated three times. The embryos and larvae from the four different groups were collected for locomotor behavior assays and whole-mount in situ hybridization (WISH).

### 2.8. Quantitative Real-Time PCR (qRT-PCR)

In this study, 30–50 larvae at 96 hpf per sample were collected for RNA extraction and used to detect the transcription levels of the representative genes in *slc6a4a^−/−^* at different developmental stages. The total RNA for each sample was extracted using 1 mL Trizol reagent (Invitrogen, Carlsbad, CA, USA) and was reverse-transcribed to cDNA using an M-MLV Reverse-Transcript Kit (Applied Biological Materials, Inc., Richmond, BC, Canada). Quantitative real-time PCR (qRT-PCR) was conducted in a CFX Connect Real-Time PCR Detection System (Bio-Rad Laboratories, Hercules, CA, USA) using iQTM SYBR Green Super mix (Bio-Rad Laboratories, Hercules, CA, USA). Thermal cycling conditions were 95 °C for 30 s, followed by 40 cycles of 95 °C for 5 s and 60 °C for 30 s. A melt curve analysis from 65 to 95 °C was performed to confirm amplification specificity. Primer sequences for qRT-PCR are mentioned in Supplementary Table S2. All experiments were repeated three times, using *18s* as an internal control.

### 2.9. Whole-Mount In Situ Hybridization (WISH)

To study gene transcription levels and their spatial distribution in the whole embryo, digoxigenin (DIG)-labeled antisense RNA probes were designed and synthesized using Sp6 or T7 transcription polymerase and a DIG RNA labeling kit (Roche Molecular Biochemicals, Penzberg, Germany) [16]. The antibody used in whole-mount in situ hybridization (WISH) was anti-dig-AP Fab fragments (Roche Molecular Biochemicals). In the probe labeling embryos, positive purple signals in cells labeled the targeted gene transcripts, and the intensity indicated the relative transcription level of the target gene. Embryos were observed and photographed under a stereoscopic microscope (Leica M205FA, Wetzlar, Germany). Using this technique, we quantified the specific distribution and relative transcription levels of the tested genes in whole embryos or larvae. Embryos or larvae with weaker purple staining were defined as embryos or larvae with reduced gene expression when compared to the signals in embryos and larvae in the control group. The percentage with reduced gene transcripts was calculated using the formula P = N_reduced_/N_total_. The number of embryos or larvae displaying reduced probe staining was represented by N_reduced_, and N_total_ indicated the total number of embryos or larvae used for probe staining in each test. All primer sequences for WISH probes are listed in Supplementary Table S3. In this study, WISH was performed on at least 15–20 embryos from each group in each biological repeat. Two or three biological repeats were performed for WISH assays in this study.

### 2.10. Frozen Section

Zebrafish larvae at 96 hpf were fixed with 4% paraformaldehyde (PFA) at 4 °C overnight and then washed three times with phosphate-buffered saline (PBS) at room temperature (RT). Next, the samples were immersed in phosphate-buffered saline (PBS) with 30% sucrose at RT for 2 h. The permeated embryos were embedded in TissueTek optimum cutting temperature (O.C.T.) compound (Sakura Finetek, Torrance, CA, USA) for cryosectioning at 6–8 μm in thickness using a frozen microtome (Thermo Fisher Scientific, Waltham, MA, USA). The sections were stored at 4 °C. After drying, the sections were used for hematoxylin and eosin (H&E) staining, immunofluorescence (IF), and terminal deoxynucleotidyl transferase-mediated dUTP-biotin nick end labeling (TUNEL) assays.

### 2.11. H&E Staining

After rapid rinsing with distilled water at room temperature (RT) for 2 min, the cryo-sections were stained with filtered 0.1% hematoxylin solution. Following a rapid rinse in distilled water for 30 s, the sections were treated with 1% acid alcohol, rinsed with water for 4 min, and then counterstained in 0.5% eosin for about 30 s at RT. Following a 3-min rinse with water, the sections were gradually dehydrated in 95 and 100% ethanol and then in xylene three times, for 15 min each. Finally, the H&E-stained sections were observed and photographed under a microscope (ZEISS Axio Imager A2, Oberkochen, Germany) to obtain high-resolution images.

### 2.12. Immunofluorescence and TUNEL Assays

The immunofluorescence (IF) assays were performed with the primary antibodies against cleaved Caspase-3 (A0214, ABclone, Woburn, MA, USA, 1:200), PH-3 (AF3358, Affinity, San Francisco, CA, USA, 1:200), Opn1sw2 (Azb21565b, Abcepta, San Diego, CA, USA, 1:200), Sox2 (A0561, ABclone, 1:200), and the fluorescent secondary antibodies (AS053, ABclone, 1:500; AS058, ABclone, 1:500, conjugated with Alexa Fluor 488 or 599, respectively) at 37 °C for 2 h. For copper-ion co-staining, sections were stained with the Cu probe (Rhodamin fluorescence, labelling intracellular Cu^+^ [14]) (provided by Prof. Gao, Hebei University, Baoding, 071002, China). The copper probe was diluted at 1:200 in BSA and applied to eye sections for 7 min in the dark at room temperature in this study. 4,6-Diamidino-2-phenylindole (DAPI) was used to label nuclei.

TUNEL apoptosis detection was performed with a TUNEL detection kit (Abbkine kit, Cat No. KTA2010, Wuhan, China) following a previously described protocol [21].

In this study, reactive oxygen species (ROS) levels in WT and *slc6a4a^−/−^* larvae were calculated using a DCFH-DA (2′,7′-dichlorodihydrofluorescein diacetate) Reactive Oxygen Species Assay Kit (S0033S, Beyotime, Shanghai, China), which was performed following the manufacturer’s instructions [22]. DCFH-DA labelled embryos were photographed under a stereoscopic microscope (Leica M205FA).

The images of cell apoptosis and immunofluorescence (DCFH-DA or copper probe positive) were obtained with a confocal microscope (Olympus FV1000 Confocal Microscope A1 HD25, Kyoto, Japan) using NIS-Elements software version 5.30.02 with a 10× objective lens with a numerical aperture of 0.45. The pinhole size was maintained at 65.70 μm, and images were collected at 1024 × 1024 pixels with a 16-bit depth. For each experiment, the same excitation/emission channel settings, laser power, detector gain, scanning speed, exposure/acquisition time, and image resolution were kept constant between WT and *slc6a4a^−/−^*.

### 2.13. Western Blotting (WB) Analysis

Samples of embryos and larvae were homogenized in radioimmunoprecipitation assay (RIPA) lysis buffer containing a protease inhibitor (Cat No. 89900, 1:1000, Thermo Fisher Scientific). The protein sample was boiled for 13 min after the addition of SDS-PAGE loading buffer, and the samples with equal amounts of protein (20 μg) were used for polyacrylamide gel electrophoresis. The separated proteins in each lane were transferred to a polyvinylidene fluoride (PVDF) membrane (Bio-Rad Laboratories) for the following Western blotting (WB) assays. The blots were blocked with 5% skim milk in Tris-buffered saline (TBS) containing 0.1% Triton X-100, followed by incubation first with the primary antibodies, Atp7b (A5676, Abclone, 1:200), and Atf4 (10835-1-AP, Proteintech, Rosemont, IL, USA, 1:1000), Calnexin (AF5362, Affinity, 1:1000), Sox2 (A0561, Abclonal, Woburn, MA, USA, 1:200), Gapdh (A19056, Abclone, 1:50,000), β-Actin (AC026, Abclone, 1:10,000), and then with secondary antibodies Goat anti-Rabbit lgG(H + L) (Cat# BL033A, Biosharp) in a 1:1000 dilution. Finally, the blots were visualized using enhanced chemiluminescence (Bio-Rad Laboratories, Hercules, CA, USA). The protein levels were quantified based on the band density using Multi Gauge V3.0.

### 2.14. Statistical Analysis

For WISH and IF analyses, more than 10 embryos or larvae were used per group (*n* > 10) in each biological repeat. For the behavior experiment, 12 larvae per group were used for the experiment in each biological repeat. Fifty embryos were used per group for RNA or protein extraction. Each assay was performed with two or three biological replicates. The statistical data of whole-mount in situ hybridization were determined by hypergeometric distribution analysis using the software R-console version 4. 4. 2 (https://www.r-project.org/). Statistical data of the signal area in different samples were analyzed with ImageJ 1.46r, and data were analyzed by GraphPad Prism 8.2.1 software. Data were expressed as the mean ± SD. Comparisons between two independent groups were performed using an unpaired, two-tailed Student’s *t*-test. One-way ANOVA following the Tukey test was used among more than two groups. Experiments involving two independent factors were analyzed using two-way ANOVA, followed by Tukey’s multiple comparisons test. The statistical significance between groups was determined at *p* < 0.05 (*), *p* < 0.01 (**), or *p* < 0.001 (***).

## 3. Results

### 3.1. slc6a4a Deficiency Induces Eye Developmental Defects and Dysfunctional Locomotor Behavior in Zebrafish

**Figure 1:**
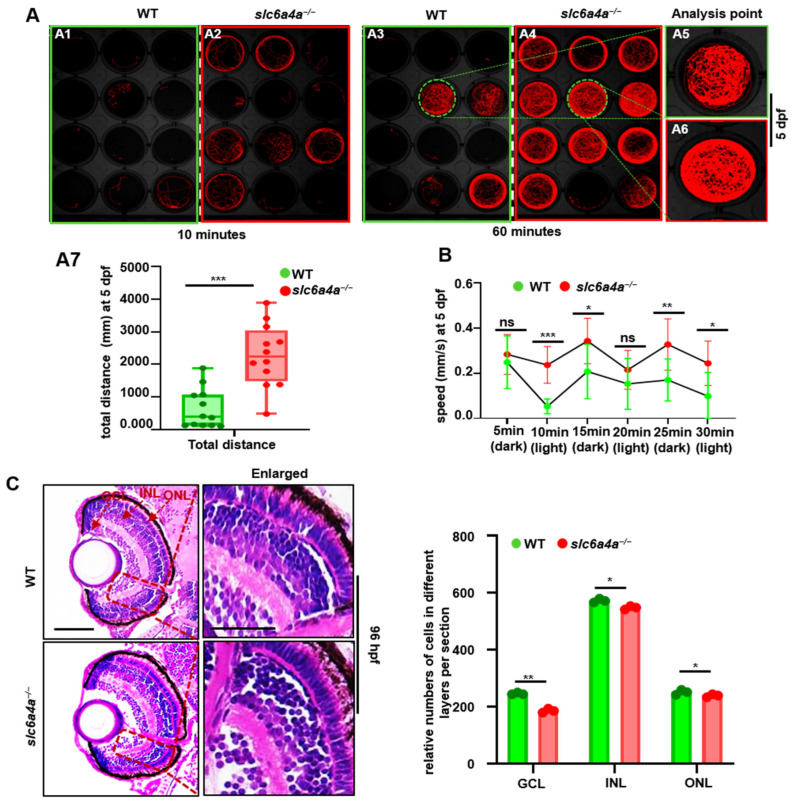
The *slc6a4a* mutation leads to abnormal locomotion and retinal defects. (**A**) Representative image showing behavioral assays of WT and *slc6a4a^−/^^−^* larvae in 10 min (**A1**,**A2**, left) and 60 min (**A3**,**A4**, right) at 5 dpf in behavioral Danio vision monitoring tests. The swimming patterns of WT (**A5**) and *slc6a4a^−/−^* (**A6**) larvae were magnified to show differences in behavioral activity. (**A7**) shows the total distance traveled. (**B**) Maximum speed (mm/s) during each 5-min cycle of the dark/light stimulation. (**C**) Hematoxylin & Eosin (HE) staining showing differences between eyes of WT and *slc6a4a^−/−^* larvae at 96 hpf (**C**, left); ONL stands for outer nuclear layer, INL for inner nuclear layer, and GCL for ganglion cell-layer, and quantification shows the number of cells in each layer in the red boxes (**C**, right). Data are presented as mean ± SD. * *p* < 0.05. ** *p* < 0.01, *** *p* < 0.001; ns, not significant. Scale bar, 100 μm (**C**), 25 μm (**C**, Enlarged).

Zebrafish Slc6a4a and Slc6a4b are highly evolutionarily conserved with human SLC6A4, with Slc6a4a exhibiting 81.0% similarity and Slc6a4b 74.8% to human SLC6A4 (Figure S1). We constructed a *slc6a4a* mutant (*slc6a4a^−/−^*) zebrafish line (Figure S2A), and the mutants exhibited no obvious morphological differences from their WT siblings from embryonic stages to adulthood (Figure S2B,C). However, the mutant larvae exhibited hyperactive locomotor behavior and mainly behaved in an abnormal locomotory pattern along the well wall (Figure 1A). Additionally, the maximum speed of *slc6a4a^−/−^* larvae was significantly increased after both light and dark stimulations relative to those in their WT controls (Figure 1B), further indicating that the mutants exhibited hyperactive behaviors and responses.

We then asked whether the mutants exhibited eye developmental defects as we observed in other zebrafish mutants with locomotor dysfunction [12,14]. The zebrafish eye includes the lens and retina, and the retina contains three cell layers, including the ONL (outer nuclear layer), INL (inner nuclear layer), and GCL (ganglion cell layer). H&E staining for retinal sections demonstrated significantly reduced cell numbers in the three retinal layers of *slc6a4a^−/−^* larvae at 96 hpf (Figure 1C). These results indicate that the mutants exhibit defects in retinal development, which might be the potential mechanism underlying the fixed locomotory pattern along the well wall of the mutants.

Studies suggest that serotonin signaling is tightly associated with numerous neurological and psychiatric symptoms [23,24]; we then wonder about the transcriptional expression of genes in the signaling in *slc6a4a^−/−^*. Serotonin-related marker *mao* was reduced significantly (Figure S2D), suggesting the alteration of serotonin signaling in the mutants, which might also attribute to the dysfunctional locomotor behavior in the mutants. Meanwhile, the transcription level of *slc6a4b* was increased significantly in *slc6a4a^−/−^*, suggesting that a genetic compensation response (GSR) might occur in the mutants as studies have reported [25,26].

### 3.2. slc6a4a Deficiency Leads to Copper Accumulation In Vivo

**Figure 2:**
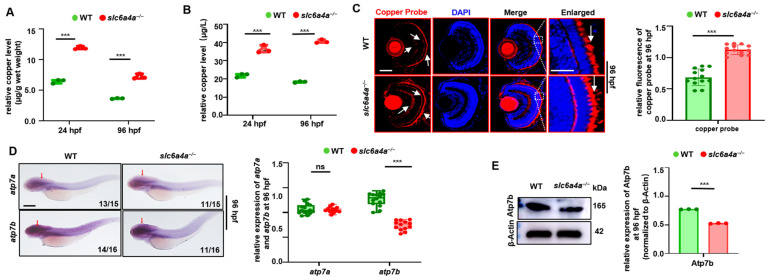
*slc6a4a* deficiency causes copper overload and changes in copper transporter gene expression. (**A**,**B**) Quantification of the copper level in whole embryos at 24 and 96 h post-fertilization (hpf). (**A**) reveals the copper content calculated according to wet weight (µg/g), and (**B**) displays the copper concentration (µg/L). (**C**) Immunofluorescence (IF) was used to detect the relative copper ion level in sectioned eyes using copper probes in WT and *slc6a4a^−/^^−^* larvae at 96 hpf (**C**, left), white arrows indicate copper probe signals in the retinal region, with calculation of the copper relative fluorescence (**C**, right). (**D**) Transcription level of copper transporter genes *atp7a* and *atp7b* at 96 hpf in WT and *slc6a4a^−/^^−^* (**D**, left), red arrows indicate the expression of gene signals, with calculation of the relative expression levels of *atp7a* and *atp7b* (**D**, right). (**E**) Western blotting (WB) analysis of Atp7b at 96 hpf in WT and *slc6a4a^−/^^−^* larvae (**E**, left), with calculation of the relative expression levels of Atp7b (**E**, right). Data are analyzed using GraphPad Prism 8.2.1, presented as mean ± SD. *** *p* < 0.001, ns, not significant. Scale bar, 50 μm (**C**), 25 μm (**C**, Enlarged), 200 μm (**D**).

It has been reported that human HeLa cells with *SLC6A4* deficiency exhibit increased intracellular copper levels [10]. Thus, we measured total copper levels in WT and *slc6a4a^−/−^* embryos at 24 hpf and 96 hpf, respectively, using ICP-MS to determine whether *slc6a4a* mutation is also linked with copper homeostasis in an in vivo zebrafish model. Analysis revealed a significantly (*p* < 0.001) higher copper content in *slc6a4a^−/−^* embryos and larvae, compared with that in the WT group at both 24 hpf and 96 hpf, and the tendency was consistent using two standard calculation methods (μg/g, μg/L) (Figure 2A,B). Additionally, significant copper-ion accumulation was observed (red fluorescence, staining with copper probes [14]) in the retina in *slc6a4a^−/−^* mutants (Figure 2C).

Atp7a/7b are important copper-transporting ATPases, especially in copper efflux in cells in vertebrates [27]. We then observed that there were no significant differences in the transcription levels of the copper transport gene *atp7a* in *slc6a4a^−/−^* mutants, compared with its levels in WT. However, the *atp7b* transcription level exhibited a significant reduction in the mutants (Figure 2D). The transcripts of *atp7b* in the brain (indicated via red arrows) were significantly reduced in *slc6a4a^−/−^*. Consistently, the Atp7b protein level was significantly reduced in the mutant at 96 hpf (Figure 2E). These results indicate that the mutants exhibit a reduction in Atp7b levels, which might be attributed to the consequent impaired copper efflux and copper overload in the mutants.

### 3.3. slc6a4a Deficiency Is Associated with Elevated ROS and ER Stress and Retinal Cell Apoptosis

**Figure 3:**
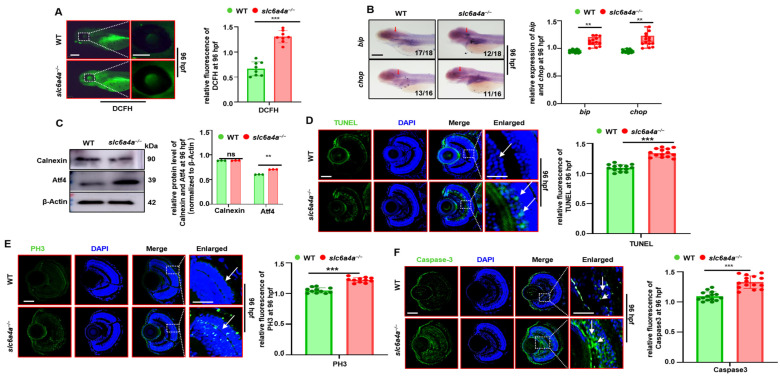
*slc6a4a* deficiency leads to activation of ROS, ER stress, and retinal cell apoptosis. (**A**) Reactive oxygen species (ROS) levels in the WT and *slc6a4a^−/−^* at 96 hpf (**A**, left), with quantification of the relative DCFH fluorescence level (labelling ROS) (**A**, right). (**B**) Transcriptional expression of *bip* and *chop* in WT and *slc6a4a^−/−^* at 96 hpf larvae (**B**, left), red arrows represents gene signals, with calculation of the relative transcription levels of genes *bip* and *chop* (**B**, right). (**C**) WB detection of Calnexin and Atf4 in the WT and *slc6a4a^−/−^* larvae at 96 hpf (**C**, left), with calculation of the relative protein levels of Calnexin and Atf4 (**C**, right). (**D**) Cell apoptosis by TUNEL staining in eye sections from WT and *slc6a4a^−/−^* at 96 hpf (**D**, left), with calculation of the relative fluorescence intensity (**D**, right). (**E**) Cell proliferation assay by PH3 staining (green dots) in eye sections from WT and *slc6a4a^−/−^* larvae at 96 hpf (**E**, left), with measurement of the relative fluorescence intensity (**E**, right). (**F**) Immunostaining of Caspase3 (green) in eye sections from WT and *slc6a4a^−/−^* larvae at 96 hpf (**F**, left), with calculation of relative intensity of the fluorescence (**F**, right). (**D**–**F**) white arrows shows the fluorescence signals. Data are presented as mean ± SD. ** *p* < 0.01, *** *p* < 0.001; ns, not significant. Scale bar, 200 μm (**A**,**B**), 50 μm (**D**–**F**), 25 μm (**A**,**D**–**F**, Enlarged).

Copper overload has been reported to trigger ROS and endoplasmic reticulum (ER) stress in zebrafish, resulting in retinal cell apoptosis during embryogenesis [13,14]. Thus, we measured ROS and ER responses in *slc6a4a^−/−^* mutant embryos and larvae. A marked increase in ROS was observed in the retina in *slc6a4a^−/−^* mutants (Figure 3A). Meanwhile, ER stress indicators *bip* and *chop* [28] were upregulated in the *slc6a4a^−/−^* embryos and larvae, as compared with their expression in WT (red arrows indicate the brain) (Figure 3B and Figure S2E). Consistently, the ER stress marker, protein Atf4, was markedly upregulated, while the Calnexin protein was unchanged in the whole *slc6a4a^−/−^* larvae at 96 hpf (Figure 3C). Together, these results indicate that *slc6a4a* deficiency leads to elevated ROS and ER stress during embryogenesis.

ROS and ER stress are triggers of cell apoptosis, and we also wonder whether reduced proliferation or increased apoptosis attributes to the reduction in retinal cells in the *slc6a4a^−/−^* mutants. We observed an increase in both apoptosis (TUNEL assays) (Figure 3D) and proliferation (PH3 staining) (Figure 3E) in retinal cells in the mutants at 96 hpf, accompanied by an increase in apoptosis inducer Caspase-3 in the cells (Figure 3F). These results demonstrate that increased cell apoptosis rather than cell proliferation contributes to the reduction in retinal cells in *slc6a4a^−/−^* mutants.

### 3.4. slc6a4a Deficiency Impairs Formation of Opsin and Rhodopsin Cells

**Figure 4:**
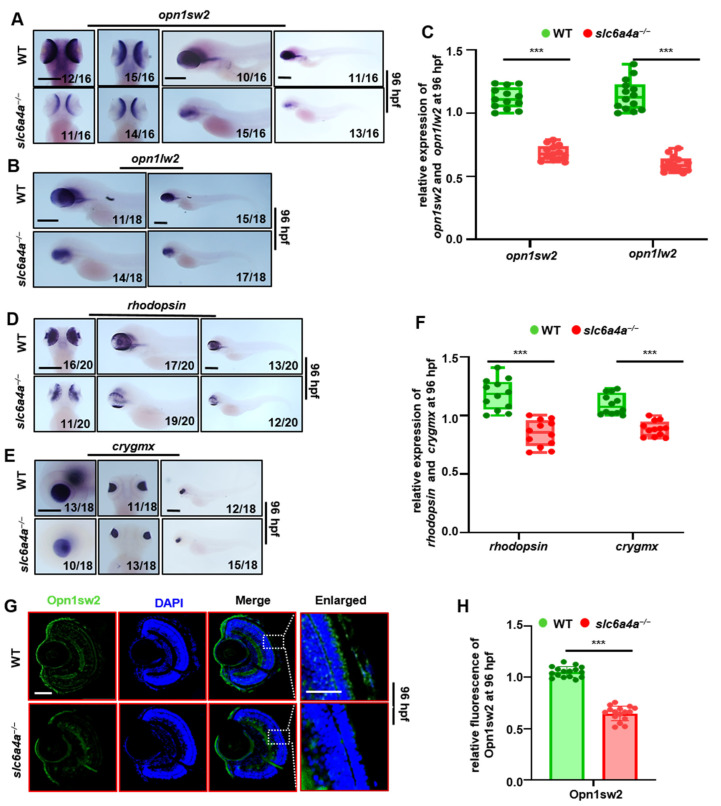
*slc6a4a* deficiency leads to retinal defects. (**A**,**B**) WISH analysis of *opn1sw2* and *opn1lw2* in WT and *slc6a4a^−/−^* larvae at 96 hpf (**A**,**B**, left). (**C**) Quantification of the relative transcription levels of *opn1sw2* and *opn1lw2*. (**D**,**E**) Transcription level of genes *rhodopsin* and *crygmx* in WT and *slc6a4a^−/−^* larvae at 96 hpf, respectively (**D**,**E**). (**F**) Quantification of their relative transcription levels. (**G**) Immunostaining of Opn1sw2 (labelling cone photoreceptors) in eye sections of WT and *slc6a4a^−/−^* larvae at 96 hpf (**G**, left). (**H**) Quantification of the relative fluorescence intensity. Data are presented as mean ± SD. *** *p* < 0.001. Scale bar, 200 μm (**A**–**E**), 50 μm (**G**), 25 μm (**G**, Enlarged).

We next tested the transcription and translation levels of retinal cone and rod cells in *slc6a4a^−/−^* to assess the specification, differentiation, and maintenance of the retinal cells. The transcription level of cone cell markers (*opn1sw2* and *opn1lw2*) (Figure 4A–C), rod cell marker (*rhodopsin* (*rho*)), and photoreceptor cell marker (*crygmx*) (Figure 4D–F) were all reduced in *slc6a4a^−/−^* embryos and larvae through WISH assays. qRT-PCR assays also uncovered the significant reduction in transcription levels of photoreceptor genes *crygm5*, *crygm2c*, *cryba1b*, *cryba2a*, *cryba4*, and *cryba2b* (Figure S2F), and eye markers *rho* and *elovl2* (Figure S2G) in *slc6a4a^−/−^* embryos and larvae. *pde6ha*, which encodes a cone-specific phosphodiesterase enzyme in phototransduction [29], was unaltered at the transcription level in *slc6a4a^−/−^* mutants (Figure S2G). Moreover, a relative reduction was observed in cone cell marker Opn1sw2 in eyes of the *slc6a4a^−/−^* at 96 hpf by immunofluorescence analysis (Figure 4G), and an obvious reduction in the immune-positive signal for protein Opn1sw2 (green) was observed in *slc6a4a^−/−^* retinas (Figure 4G,H).

### 3.5. slc6a4a Deficiency Impairs Retinal Progenitor Maintenance

**Figure 5:**
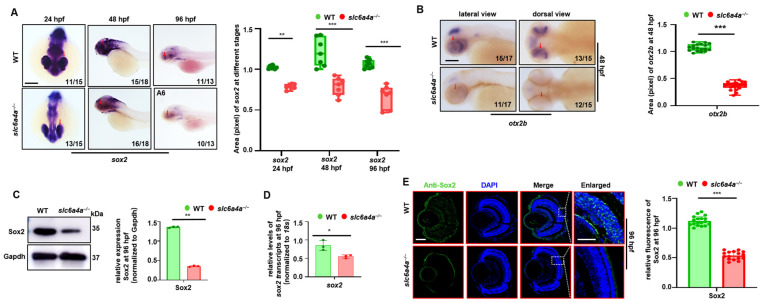
*slc6a4a* deficiency impairs the maintenance of retinal progenitor cells. (**A**) Expression levels of *sox2* at 24, 48, and 96 hpf in WT and *slc6a4a^−/^^−^* embryos and larvae (**A**, left), with calculation of the relative transcription levels of *sox2* (**A**, right). (**B**) Transcription level of *otx2b* in WT and *slc6a4a^−/^^−^* at 48 hpf in embryos, with lateral and dorsal view (**B**, left) and calculation of the relative expression (**B**, right). (**A**,**B**) red arrows indicate gene expressions. (**C**) WB analysis of the protein level of Sox2 in WT and *slc6a4a^−/^^−^* larvae at 96 hpf, with Gapdh as the internal control (**C**, left) and quantification of the protein level (**C**, right). (**D**) Quantitative PCR of the *sox2* gene and the relative levels. (**E**) Immunostaining for Sox2 in the eye sections of WT and *slc6a4a^−/^^−^* larvae at 96 hpf (**E**, left), with evaluation of the relative fluorescence intensity (**E**, right). Data are presented as mean ± SD. * *p* < 0.05. ** *p* < 0.01, *** *p* < 0.001. Scale bar, 200 μm (**A**,**B**), 50 μm (**E**), 25 μm (**E**, Enlarged).

Neural precursors are the source of all mature neurons in the retina, and retinal stem and progenitor cells (RSPCs) are located in the retina’s outermost region, which is considered as a stem-cell niche and positive for Sox2 expression [30,31]. A significant reduction was observed in the transcriptional expression of *sox2*, marking RSPCs and retinal progenitors [32], in *slc6a4a^−/−^* at different developmental stages from 24 hpf to 96 hpf (Figure 5A,D). Similarly, transcription level of retinal progenitor marker *otx2b* [33] was also reduced in *slc6a4a^−/−^* (Figure 5B). Additionally, the protein level of Sox2 was decreased obviously in the whole larvae of *slc6a4a^−/−^* as compared with its level in the WT control (Figure 5C), and the fluorescence intensity of the Sox2 positive signal was markedly decreased in retinal cells in *slc6a4a^−/−^* at 96 hpf, especially in the outermost retinal region (Figure 5E). Collectively, the above results demonstrate that *slc6a4a^−/−^* embryos have reduced retinal progenitor cells, which reflect impaired progenitor maintenance rather than reduced proliferation because we have observed the increase in proliferation signals of PH3 in the retina in the mutants.

### 3.6. atp7b mRNA Partially Rescues Retinal Defects in slc6a4a^−/−^ Larvae

We next wonder the specificity of locomotor and retinal defects in *slc6a4a^−/^^−^* larvae. Notably, ectopic expression of full-length *slc6a4a* mRNA not only partially recovered the dysfunctional locomotor activity (Figure S3A) but also recovered the transcription levels of retinal markers in *slc6a4a^−/^^−^* larvae (Figure S3B), supporting that *slc6a4a* deficiency induces the defects specifically.

**Figure 6:**
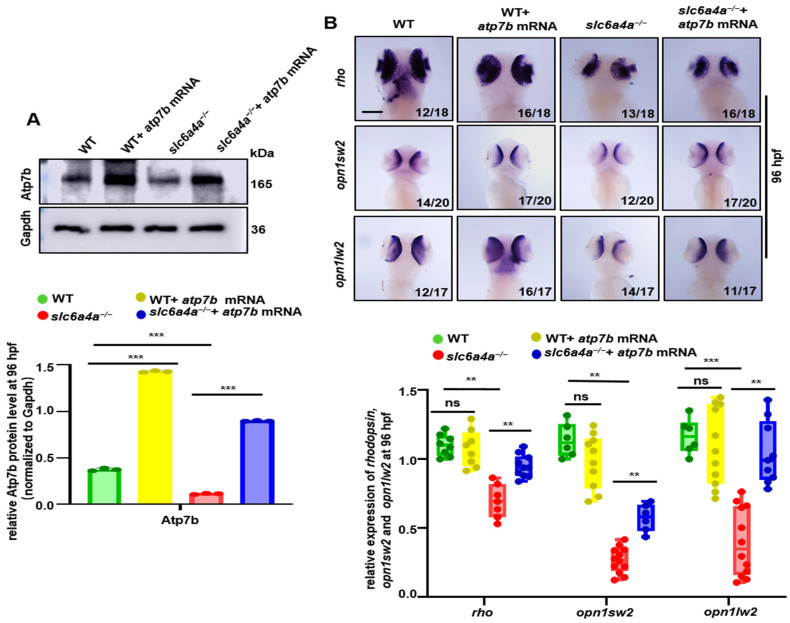
*atp7b* mRNA partially restores retinal marker expression in *slc6a4a^−/−^* larvae. (**A**) WB (**A**, up) and quantification of Atp7b protein at 96 hpf (**A**, down) in groups of WT, WT + *atp7b* mRNA, *slc6a4a^−/^^−^,* and *slc6a4a^−/^^−^*+ *atp7b* mRNA. Gapdh was used as an internal loading control. (**B**) WISH images (**B**, up) and quantification (**B**, down) of retinal markers *rho*, *opn1sw2*, and *opn1lw2* at 96 hpf, of larvae from the four groups. Data are presented as mean ± SD. *** *p* < 0.001, ** *p* < 0.01; ns, not significant. Scale bar, 200 μm (**B**).

Next, the rescue experiment via ectopic expression of full-length *atp7b* mRNA was performed to determine whether the reduction in Atp7b levels contributes to defects in *slc6a4a^−/^^−^* larvae. The results showed that the decrease in Atp7b protein levels in *slc6a4a^−/^^−^* larvae was partially recovered following *atp7b* mRNA injection (Figure 6A). Meanwhile, the reduction in transcription levels of *rho*, *opn1sw2*, and *opn1lw2* was also partially rescued in *slc6a4a^−/^^−^* larvae after ectopic expression of *atp7b* mRNA (Figure 6B). These findings suggest that retinal abnormalities associated with *slc6a4a* deficiency are functionally influenced by Atp7b downregulation. However, this study still lacks evidence to support how *slc6a4a* deficiency induces the downregulation of Atp7b.

### 3.7. Copper Chelation with TTM Partially Rescues the Abnormal Locomotor Behavior and Retinal Defects of slc6a4a^−/−^

*slc6a4a^−/−^* larvae at 5 dpf exhibited dysfunctional locomotor behavior (Figure 1); then, we asked whether *slc6a4a* deficiency-induced copper overload contributes to the defects. Tetrathiomolybdate (TTM) is a selective and efficient copper chelator [34] and effectively treats Wilson’s disease, which is characterized by a high concentration of copper in the brain and liver [35,36]. Here, we found that TTM at the concentration used in this study (200 μM) had no influence on swimming behavior (Figure S4A) or retinal gene expression (Figure S4B) in WT larvae, indicating that TTM at the used concentration itself could not alter the general locomotor activity and retinal gene expressions.

**Figure 7:**
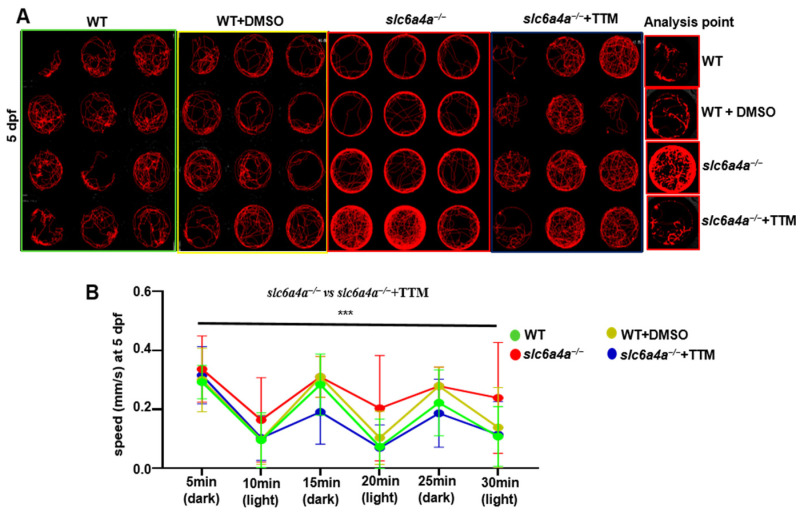
Copper chelation (TTM) partially rescues behavioral movement defects in *slc6a4a^−/^^−^*. (**A**) Locomotor tracking pattern of 5 dpf larvae from WT, WT + DMSO, *slc6a4a^−/^^−^,* and *slc6a4a^−/−^* + TTM groups. TTM treatment improves the locomotor pattern of *slc6a4a^−/^^−^* larvae, and red traces indicate swimming routes throughout the monitoring period. (**B**) Quantification of maximum speed (mm/s) at alternating 5 min dark and light intervals over 30 min. Data are presented as mean ± SD. *** *p* < 0.001; ns, not significant.

However, TTM at this concentration partially rescued the abnormal locomotory behavior around the cell wall in *slc6a4a^−/−^* larvae (Figure 7A) and partially recovered the hyperactive responses of *slc6a4a^−/−^* larvae to both light and dark stimulations (Figure 7B). Together, these results suggest that *slc6a4a* deficiency-induced copper overload partially contributes to the dysfunctional locomotor behavior of *slc6a4a^−/−^* larvae.

**Figure 8:**
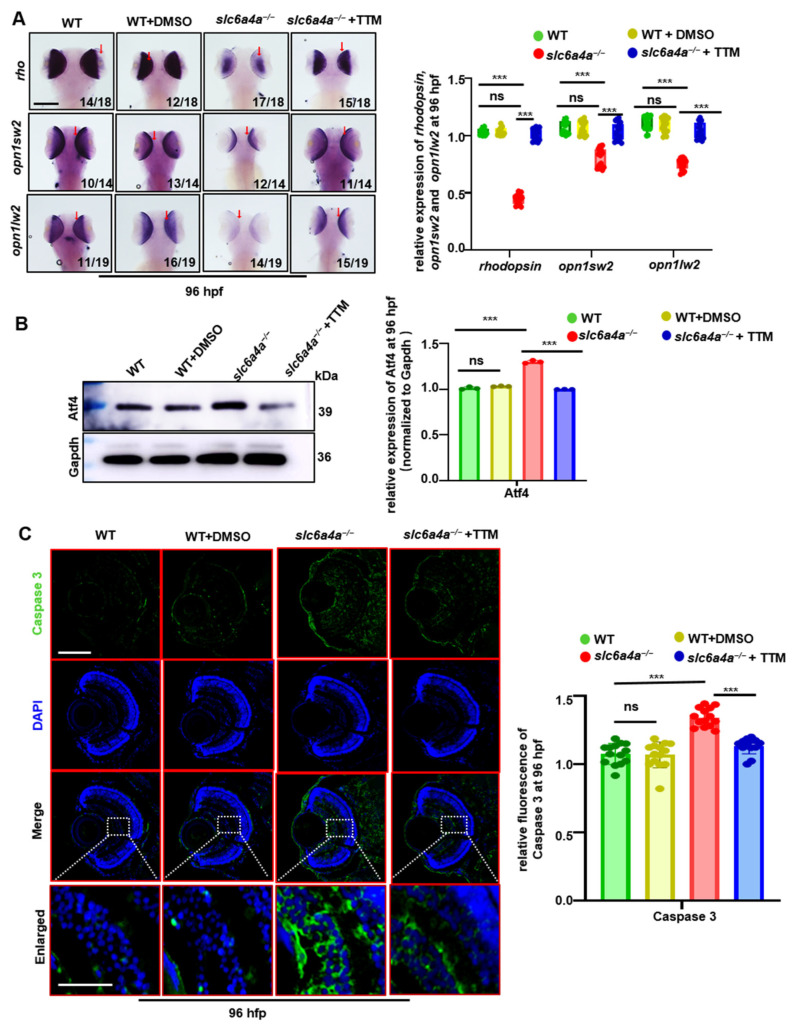
Copper chelator TTM recovers retinal defects, ER stress, and apoptosis. (**A**) Transcriptional expression of the retinal markers *rho*, *opn1sw2,* and *opn1lw2* in the WT, WT + DMSO, *slc6a4a^−/^^−^*, and *slc6a4a^−/^^−^* + TTM larvae at 96 hpf (**A**, left), red arrows indicate retinal marker expression signals in the eyes, with calculation of the relative transcription levels of *rho*, *opn1sw2,* and *opn1lw2* (**A**, right). (**B**) WB analysis of Atf4 in larvae at 96 hpf from the WT, WT + DMSO, *slc6a4a^−/^^−^*, and *slc6a4a^−/^^−^* + TTM group (**B**, left), with quantification of relative translation levels of Atf4 (**B**, right). (**C**) Immunostaining analysis of Caspase3 in retinas of 96 hpf larvae from the WT, WT + DMSO, *slc6a4a^−/^^−^,* and *slc6a4a^−/^^−^* + TTM groups (**C**, left), with quantification of the relative fluorescence intensity (**C**, right). Data are presented as mean ± SD. *** *p* < 0.001; ns, not significant. Scale bar, 200 μm (**A**), 50 μm (**C**), 25 μm (**C**, Enlarged).

Meanwhile, TTM treatment also partially rescued the copper overload in retinas in *slc6a4a^−/−^* larvae (Figure S4C) and recovered the increased expression of stress indicators, *bip* and *chop* (Figure S4D), and Atf4 (Figure 8B) to nearly normal levels in the mutants. Consistently, TTM treatment partially rescued the reduced expression of retinal cell markers *rho*, *opn1lw2*, and *opn1sw2*, in the mutants (Figure 8A and Figure S4B). The increase in cell apoptosis in retinas in *slc6a4a^−/−^* larvae was also effectively recovered to a nearly normal level via co-treatment with TTM (Figure 8C). With the observations that TTM treatment does not alter the general development of WT but partially recovers the defects in *slc6a4a^−/−^*, we demonstrate that *slc6a4a* deficiency-induced copper overload leads to elevated ROS and ER stress and results in cell apoptosis and retinal defects.

## 4. Discussion

A recent in vitro screening study in HeLa cells has identified that intracellular copper is elevated when *SLC6A4* is knocked down [10], and studies have shown that SLC6A4 is tightly associated with neural system functions. In this study, we found that *slc6a4a* deficiency led to copper overload in zebrafish embryos and larvae, which resulted in elevated ROS and ER stress and the consequent retinal cell apoptosis. Meanwhile, copper chelator TTM partially recovered the defects in locomotor behavior and retinal development in *slc6a4a^−/−^*, highlighting that *slc6a4a* deficiency induces copper overload is one of the pivotal attributors underlying retinal defects and dysfunctional locomotion. To our knowledge, this study is the first to suggest a close association of Slc6a4a, copper homeostasis, and retinal development and the consequent locomotor behaviors in an in vivo vertebrate.

In this study, our results indicate that *slc6a4a* deficiency (*slc6a4a^−/−^*) larvae are hyperactive in locomotor behavior and responses, displaying fixed behavior around the cell wall. The WT siblings behave randomly. Copper chelator TTM partially rescues the dysfunctional behavior of the mutants, demonstrating that *slc6a4a* deficiency-induced copper overload is tightly associated with the abnormal circular swimming behavior.

Studies have revealed that serotonin and serotonin receptors function in multiple brain loci, and their mutations have been reported to be lightly linked to the etiology of numerous neurological and psychiatric symptoms [23,24], including various kinds of behavior defects influenced by brain’s serotonin (5-HT) system [37,38]. This study reveals the altered expression of several genes in the serotonin system in *slc6a4a^−/−^*. This not only suggests that altered serotonin signaling might be attributed to the abnormal swimming behavior of the mutants but also suggests that the mutants are ideal models to test the causal relationship of *slc6a4a* deficiency, serotonin (5-HT) system, and behavior defects in a future study. Additionally, it has been reported that the SLC6A4 methylation status is tightly associated with symptoms of attention deficit hyperactivity disorder (ADHD) [39], which is characterized by impulsivity, hyperactivity, lack of attention, and behavioral anxiety [40]. Here, we observe that *slc6a4a^−/−^* is hyperactive, and we will detect whether the mutants could be used as ADHD-like models in the next project.

In this study, we reveal that *slc6a4a* deficiency induces the downregulation of Atp7b, and studies report that Atp7b deficiency leads to copper overload in cells of liver and brain, among other cells, both in vivo and in vitro [41]. We speculate that *slc6a4a* deficiency induced Atp7b downregulation might attribute to the copper overload in HeLa cells [10] and in zebrafish embryonic cells in this study. Meanwhile, the downregulation of Atp7b might not be a compensatory response because more increased expression of Atp7b is required for the efflux of overload copper in zebrafish embryonic cells in *slc6a4a^−/−^*. We speculate that the altered Serotonin signaling or the unbalanced cellular homeostasis of 5-HT might contribute to the downregulation of Atp7b in *slc6a4a^−/−^* mutants.

A recent study has revealed that zebrafish *atp7b^−/−^* larvae show copper overload and developmental defects of retinal cells and consequent dysfunctional behavior [14]. Particularly, *slc6a4a^−/−^* larvae also show developmental defects in the retina, accompanied by lower levels of the Atp7b protein and transcripts and consequent copper overload. Collectively, these results indicate that zebrafish *slc6a4a^−/−^* mutants, similar to *atp7b^−/−^*, are potential models to recapitulate the developing Kayser–Fleischer rings [42] and retinal degeneration [43] in patients with Wilson disease. Ectopic expression of *atp7b* mRNA effectively rescues retinal defects in *slc6a4a^−/−^* larvae, further indicating that *atp7b* mediates the roles of *slc6a4a* in retinal development and the consequent locomotor behavior.

Consistent with previous results, our current results also demonstrate that *slc6a4a* deficiency leads to copper overload, increases ROS and ER stress, and induces the consequent retinal cell apoptosis, as we observed in *atp7b^−/−^* mutants [14]. ROS and redox imbalance cause neurodegenerative disorders and brain cell damage [44,45]. ER stress induces apoptosis through the downstream C/EBP homologous protein (CHOP) and IRE-Caspase pathways [46]. *slc6a4a* deficiency leads to copper dysregulation and activation of ER stress markers, such as *bip* and *chop*, and Atf4. Our study reveals molecular connections among copper overload, ROS, ER stress, and the consequent neuroretinal dysfunction, highlighting the important function of *slc6a4a* in maintaining copper homeostasis and retinal development in zebrafish. Additionally, our study revealed that mutated embryos and larvae show lower expression of the retinal progenitor markers *sox2* and *otx2b*, accompanied by an increase in the PH3-positive proliferating signal. These findings suggest the impaired maintenance of neural progenitors as we observed in *pmpcb^−/−^* mutants [21].

This research highlights the critical role of *slc6a4a* in copper homeostasis in an in vivo vertebrate and in the developing retina. Future research should examine the downstream signaling pathways linking copper homeostasis and other developmental defects in *slc6a4a^−/−^* and examine serotonin signaling pathways linking neural system development and hyperactive locomotors. Multifunctional methods that target both copper chelation and ER stress mitigation may also improve retinal recovery, given the partial rescue by TTM, which offers a translational framework for *slc6a4* dysfunction-related diseases in vertebrates.

## 5. Conclusions

In zebrafish, loss of *slc6a4a* function disrupts retinal copper homeostasis, leading to intracellular accumulation of copper that triggers retinal cell apoptosis. Our findings reveal that *slc6a4a* mutation is associated with copper overload, which induces ROS-mediated cell apoptosis, leading to retinal dysfunction and consequent locomotor dysfunction in zebrafish. This study establishes a useful zebrafish model for investigating gene–copper interactions and identifies *slc6a4a* as a contributor to copper homeostasis in retinal development. These findings provide a translational framework for retinal degenerative diseases linked to copper dysregulation and suggest potential treatments that target copper homeostasis or oxidative stress protection to maintain photoreceptor function.

## Data Availability

The original contributions presented in this study are included in the article/Supplementary Material. Further inquiries can be directed to the corresponding author.

## Supplementary Materials

The following supporting information can be downloaded at: https://www.mdpi.com/article/10.3390/ani16132036/s1, Figure S1. Bioinformatic comparison and evolutionary relationship of human SLC6A4 and zebrafish Slc6a4a/Slc6a4b. Figure S2. *slc6a4a* mutant generation and qRT-PCR analysis of Serotonin, retinal and endoplasmic reticulum (ER) stress associated genes. Figure S3. *slc6a4a* mRNA injection rescues locomotor and retinal defects in zebrafish *slc6a4a^−/−^* larvae. Figure S4. TTM treatment reduces copper accumulation and partially rescues retinal and ER stress defects in *slc6a4a^−^^/^^−^* larvae. Table S1. Genotype primer list. Table S2. (qRT-PCR) primer list. Table S3. WISH primer list. File S1. Western blotting original picture.

## Abbreviations

The following abbreviations were used in this manuscript:

SERT	Serotonin transporter
ER	Endoplasmic Reticulum
hpf	Hours post-fertilization
ROS	Reactive oxygen species
WT	Wild-type
Cu	Copper
DMSO	Dimethyl sulfoxide
PBS	Phosphate-buffered saline
WISH	Whole-mount in situ hybridization
TTM	Tetrathiomolybdate
ICP-MS	Inductively coupled plasma mass spectrometry
TUNEL	Terminal deoxynucleotidyl transferase dUTP nick end labeling
PH3	Phospho-histone H3
