# Supplementary material for: Disrupted Copper Homeostasis and Impaired Retinal Development Caused by slc6a4a Deficiency in Zebrafish
Source: Animals (Basel). 2026 Jul 2;16(13):2036. doi: 10.3390/ani16132036 (PMC13359532; doi:10.3390/ani16132036)
Supplement: Supplementary file 1 [file animals-16-02036-s001.zip › Supplementry figures.pdf]

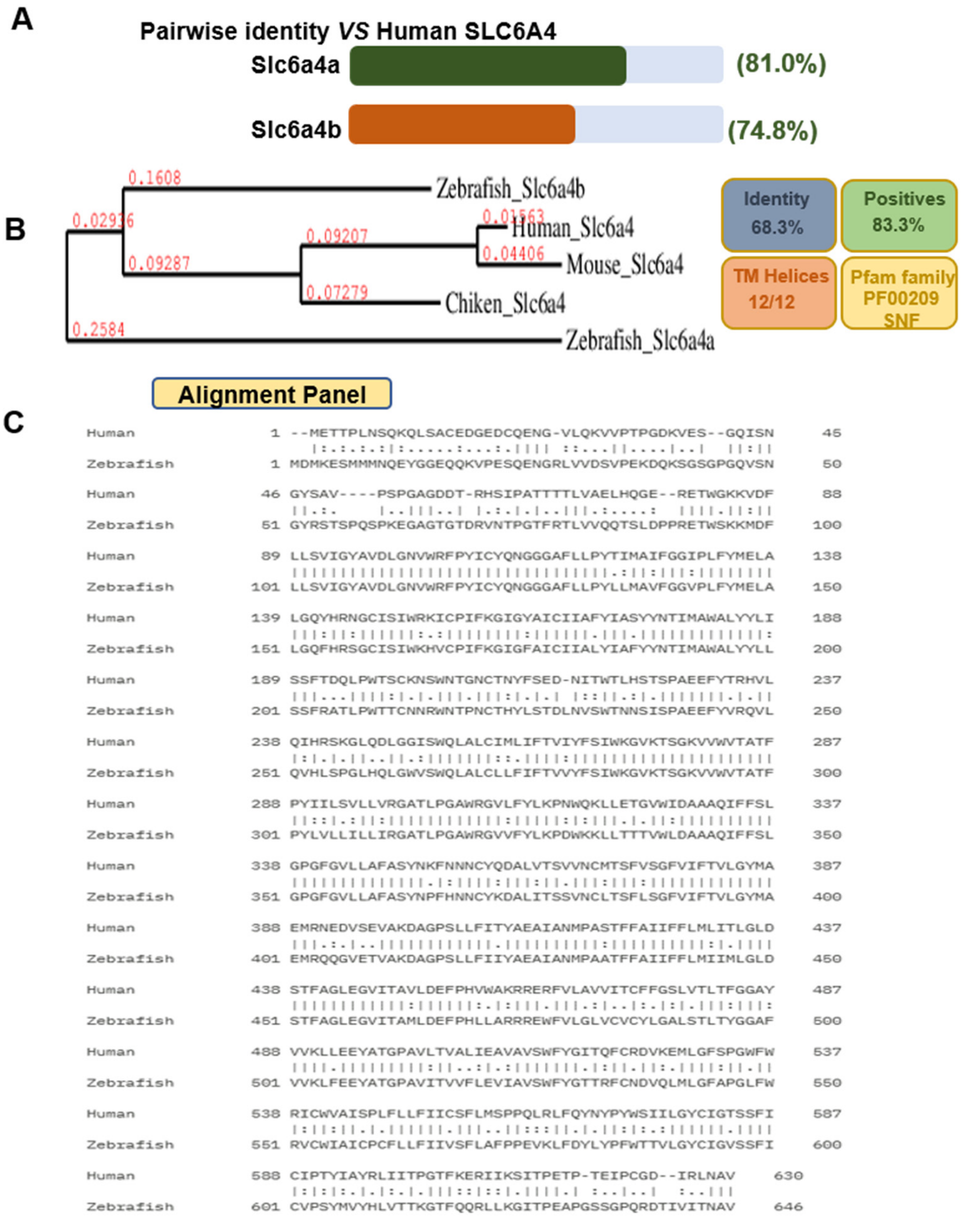

Figure S1. Bioinformatic comparison and evolutionary relationship of human SLC6A4 and zebrafish Slc6a4a/Slc6a4b. **A** shows amino acid sequence comparison, showing similarity between human SLC6A4 and zebrafish Slc6a4a/Slc6a4b. **B** representative image shows a Phylogenetic tree, showing the evolutionary relationship between human SLC6A4 and zebrafish Slc6a4a/Slc6a4b proteins. **C** shows pairwise sequence-alignment analysis, showing that zebrafish Slc6a4a/Slc6a4b are conserved homologues of human SLC6A4.

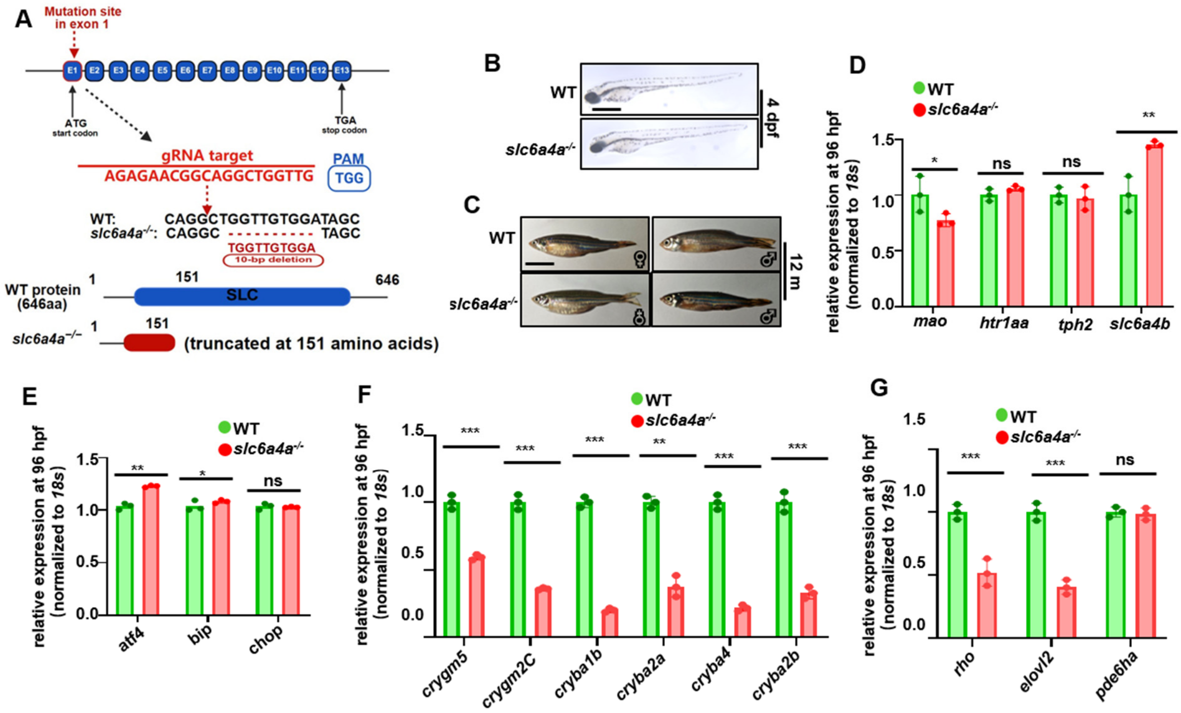

Figure S2. *slc6a4a* mutant generation and qRT-PCR analysis of Serotonin, retinal and endoplasmic reticulum (ER) stress associated genes. **A** Schematic illustration of the CRISPR/Cas9 for generation of *slc6a4a*$^{-/-}$. **B, C** represents morphological phenotype of WT and *slc6a4a*$^{-/-}$ at 4 dpf (days post fertilization) and 12 months. **D** qRT-PCR analysis of Serotonin-related genes in zebrafish larvae at 96 hpf. **E** qRT-PCR analysis of ER-stress-related genes at 96 hpf. **F** qRT-PCR analysis of crystallin and lens-related genes at 96 hpf. **G** qRT-PCR analysis of retinal and photoreceptor-related genes at 96 hpf. Data are presented as mean ± SD. * $P < 0.05$. ** $P < 0.01$, *** $P < 0.001$; ns, not significant. Scale bar, 200 μm (**B**).

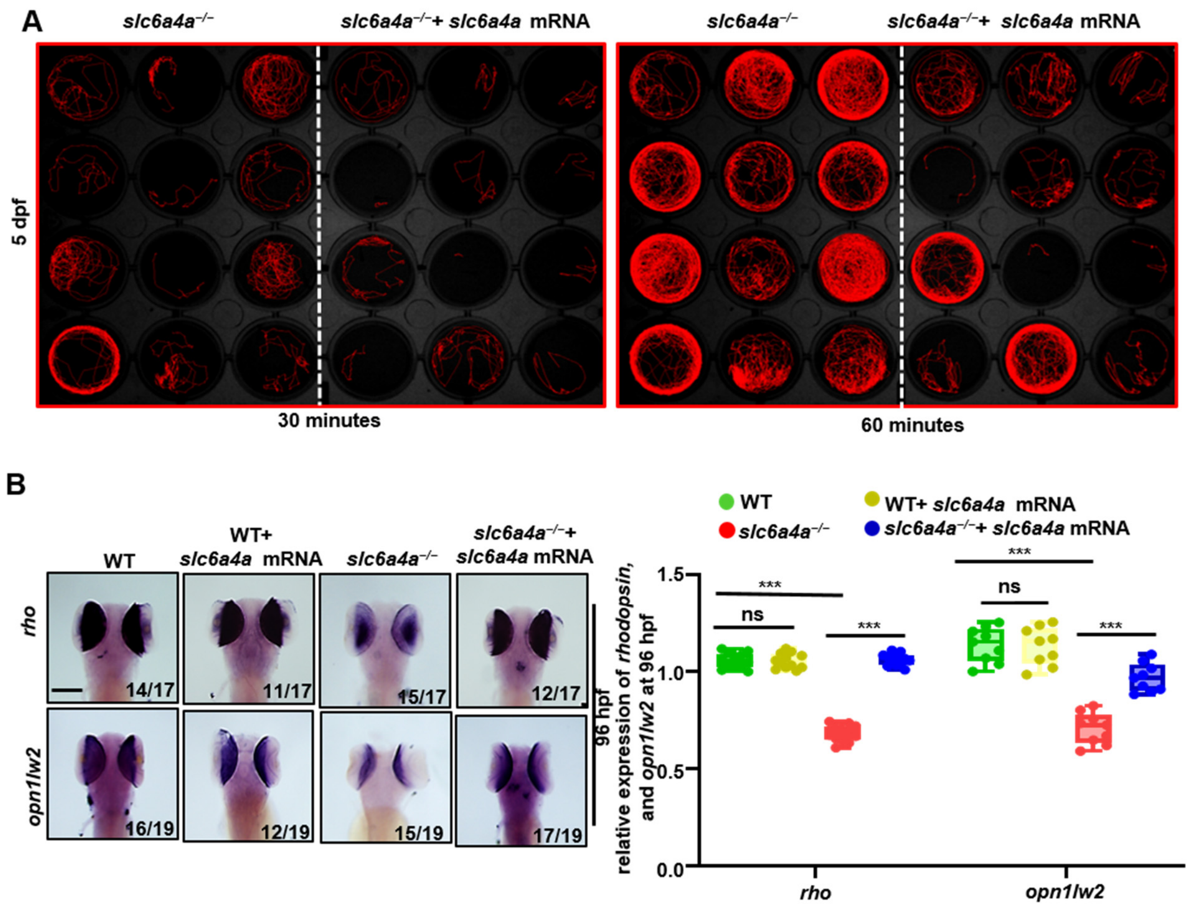

Figure S3. *slc6a4a* mRNA injection rescues locomotor and retinal defects in zebrafish *slc6a4a*$^{-/-}$ larvae. **A** locomotory trajectories of two groups, *slc6a4a*$^{-/-}$ and *slc6a4a*$^{-/-}$ + *slc6a4a* mRNA in 30 minutes total (**A,** right) and 60 minutes total (**A**, left). Red traces indicate larval movement. **B** Whole-mount *in situ* hybridization data showing transcriptional expression of retinal *rho* and *opn1lw2* (**B**, left), with quantification of WISH data (**B**, right). Data are presented as mean ± SD. * $P < 0.05$. ** $P < 0.01$, *** $P < 0.001$; ns, not significant. Scale bar, 200 μm (**B**).

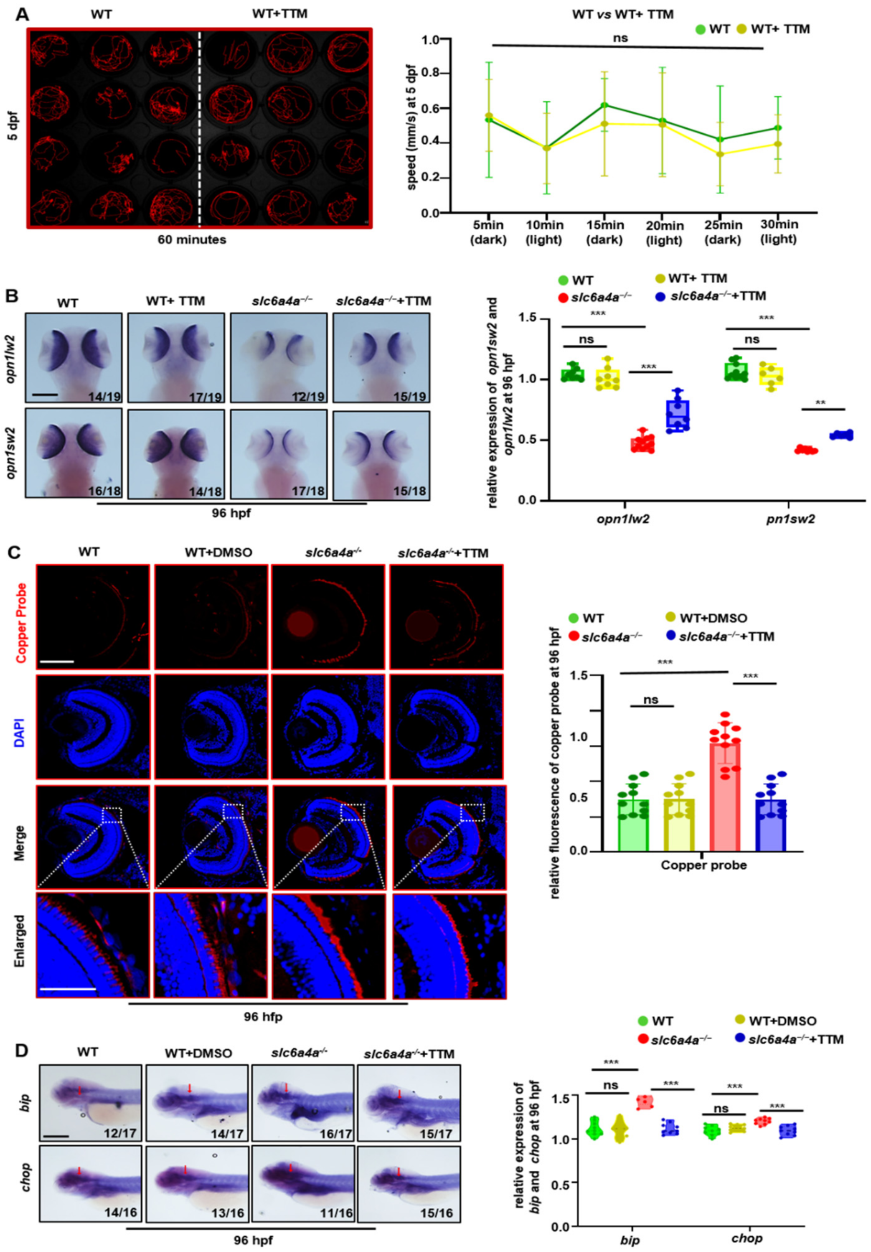

Figure S4. TTM treatment reduces copper accumulation and partially rescues retinal and ER stress defects in *slc6a4a*$^{-/-}$ larvae. **A** Representative Danio Vision behavioral activity WT and WT+TTM at 5 dfp (days post fertilization) (**A**, left), with quantification of the maximum speeds (**A**, right). **B** transcriptional expression of the retinal markers *opn1sw2* and *opn1lw2* in the WT, WT + TTM, and *slc6a4a*$^{-/-}$, *slc6a4a*$^{-/-}$ + TTM larvae at 96 hpf (**B**, left), with calculation of the relatively transcriptional levels of *opn1sw2* and *opn1lw2* (**B**, right).**C** immunofluorescence (IF) of copper ion level in sectioned eye slides using copper probes in WT, WT + DMSO and *slc6a4a*$^{-/-}$, *slc6a4a*$^{-/-}$ + TTM larvae at 96 hpf (**C**, left), with calculation of relative fluorescence of copper ions (**C**, right). **D** whole-mount *in situ* hybridization of ER stress markers *bip* and *chop* (**D**,

left), with quantification of the data (**D**, right). Data are presented as mean ± SD. * P < 0.05. ** P < 0.01, *** P < 0.001; ns, not significant. Scale bar, 200 μm (**B, D**), 50 μm (**C**), 25 μm (**C**, Enlarged),
