# Supplementary material for: Disrupted Copper Homeostasis and Impaired Retinal Development Caused by slc6a4a Deficiency in Zebrafish
Source: Animals (Basel). 2026 Jul 2;16(13):2036. doi: 10.3390/ani16132036 (PMC13359532; doi:10.3390/ani16132036)
Supplement: Supplementary file 1 [file animals-16-02036-s001.zip › Supplementry table.pdf]

Supplementary Table S1 Genotype primer list		
Gene	Full names of gene	Sequence （in 5’ to 3’ direction）
slc6a4a-F-129bp	*solute carrier family 6member 4a*	TATGAAAGAGTCGATGATGA
slc6a4a-R-129bp		GGCCCAGAGCCAGATT
slc6a4a-F-304bp	*solute carrier family 6member 4a*	TCCATCCCAGCCCACT
slc6a4a-R-304bp		TGACCCTGTCCGTCCC

Supplementary Table S2 (qRT-PCR) primer list		
Gene	Full names of gene	Sequence （in 5’ to 3’ direction）
18s-F	*18S ribosomal RNA*	CGAGCAGGAGATGGGAACC
18s-R		CAACGGAAACGCTCATTGC
crygm5-F	*Crystallin, gamma M5*	TGGACAGTCTACGCTGGAAG
crygm5-F		CAGGAGGTTGAGGAGGTTGA
crygm2c -F	*Crystallin, gamma M2c*	AGCTCTTCACCGTCTACCTG
crygm2c -R		TTGGCAGTACAGGGTGTTTG
cryba1b-F	*Crystallin, beta A1b*	CGTGAAGATGAGGAGGAGGA
cryba1b-F		CAGGATGGAGGAGGAGGAGA
cryba2a - F	*Crystallin, beta A2a*	TGGTGGTGCTGCTCTACATT
cryba2a - R		AGGATGACGATGAGGAGGAC
cryba4- F	*Crystallin, beta A4*	GCTGCTGTTGGTGACTGATT
cryba4- R		CAGGCTCAGGTTGAGGAGTT
cryba2b- F	*Crystallin, beta A2b*	CAGTCTCAGGTTGAGGAGGA
cryba2b- R		TGGAGGTTGAGGACAGGTTG
Rho- F	*Rhodopsin*	CAGGAGGAGGTTGAGGAGGT
Rho- R		CAGGAGGTTGAGGGTGAGTT
elovl2-F	*Elongation of very long chain fatty acids protein 2*	AGGCTGTTGAGGACTGGTAA
elovl2-R		CAGGAGGTTGAGGGTGAGTT
htr1aa-F	*5-hydroxytryptamine receptor 1Aa*	CTACTCAACTTTCGGGGCGT
htr1aa-R		CACCGCCAGCTATTTATCCG
Mao-F	*monoamine oxidase*	GACACAGTTGGCAAGGGTCT
Mao-R		ACTCTGTGGCCGGTTCAGTT
Slc6a4b-F	*solute carrier family 6 member 4b*	GTATTAAGAATGCTGGTTCC
Slc6a4b-R		ACTAAAGAIGCAACTTGCCA

Supplementary Table S3 (WISH primer list)		
Gene	Full names of gene	Sequence （in 5’ to 3’ direction）
opn1lw1-probe-F	*opsin 1（cone pigments），long-wave-sensitive, 1*	TGAGGGTCCCAATTACCA
opn1lw1-probe-R		TAATACGACTCACTATAGGGA GCAGATGCCCATTTAGC
opn1sw2-probe-F		CCTCGGGAACTTTACCTT

opn1sw2-probe-R	*opsin 1* （*cone pigments*） *short-wave-sensitive 2*	TAATACGACTCACTATAG GGTTGGAAACCACCCAGATT
rhodopsin-probe-F	*rhodopsin*	AACCTGGAGGGCTTCTTT
rhodopsin-probe-R		TAATACGACTCACTATAGG GTATGACTAACAGTGGGATGA
crx-probe-F	*cone-rod homeobox protein*	CGTCGTTGGGCTTCAGTTC
crx-probe-R		TAATACGACTCACTATAGGGTCT TCTTCACGCATCTTTCCTT
sox2-probe-F	*sex determining region Y-box*（*SRY*） *transcription factor 2*	CTGTCCGAGAGCGAGAAGC
sox2-probe-R		TAATACGACTCACTATAGGG GGAATGAGACGACGACGTGA
otx2b-probe-F	*orthodenticle homeobox 2b*	CTCTGTCCAACCAACACCCTAA
otx2b-probe-R		TAATACGACTCACTATAGGGGAG AAAGCCTGCCCATTCC
