# Supplementary figures and images for: Disrupted Copper Homeostasis and Impaired Retinal Development Caused by slc6a4a Deficiency in Zebrafish
Source: Animals (Basel). 2026 Jul 2;16(13):2036. doi: 10.3390/ani16132036 (PMC13359532; doi:10.3390/ani16132036)

**Fig.2 (E)**

Vazyme (MP202)

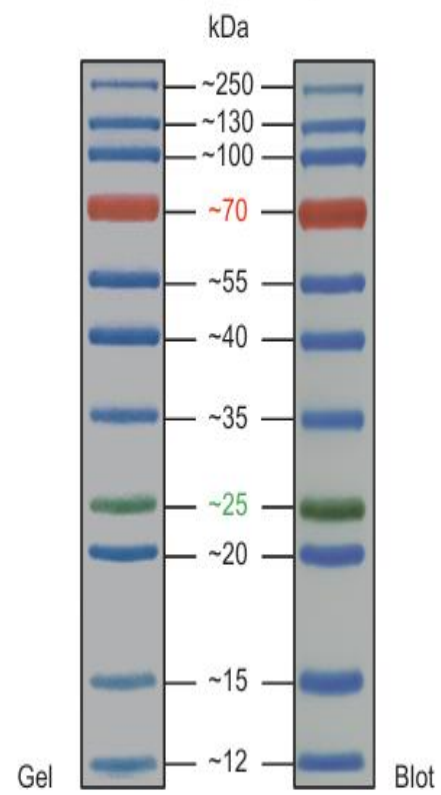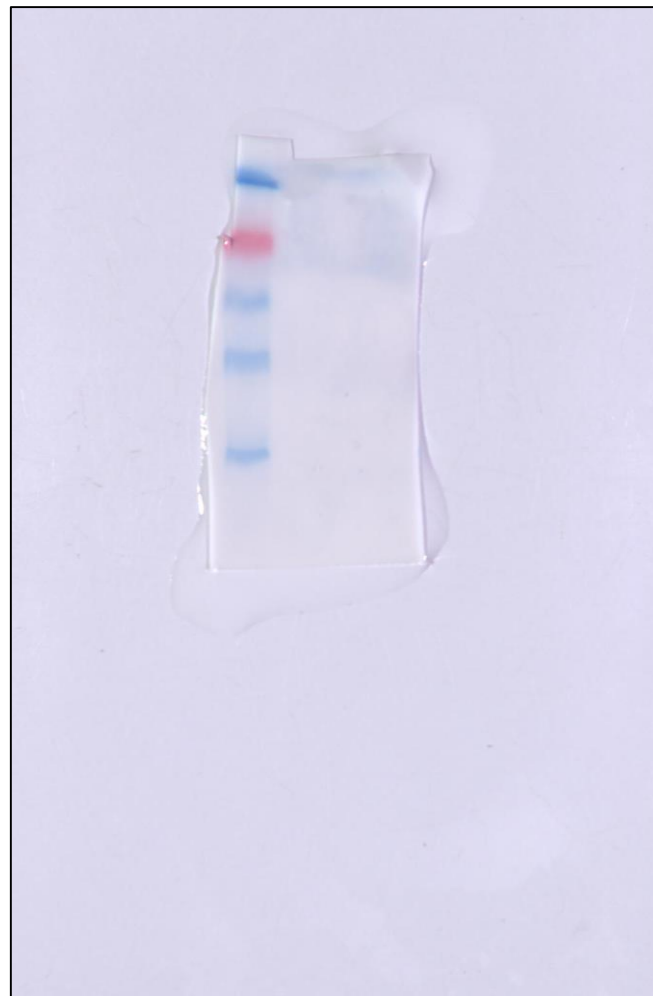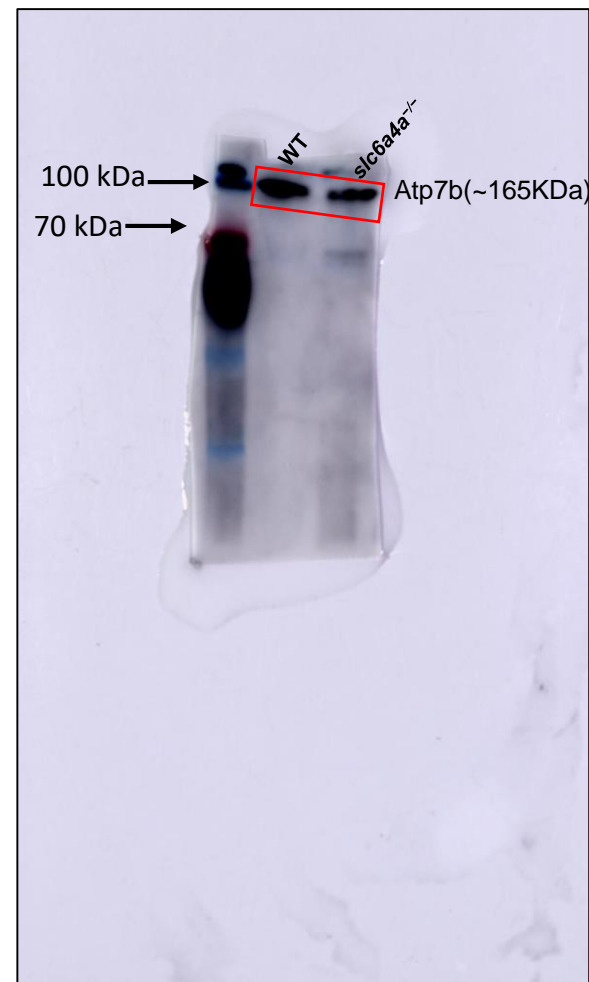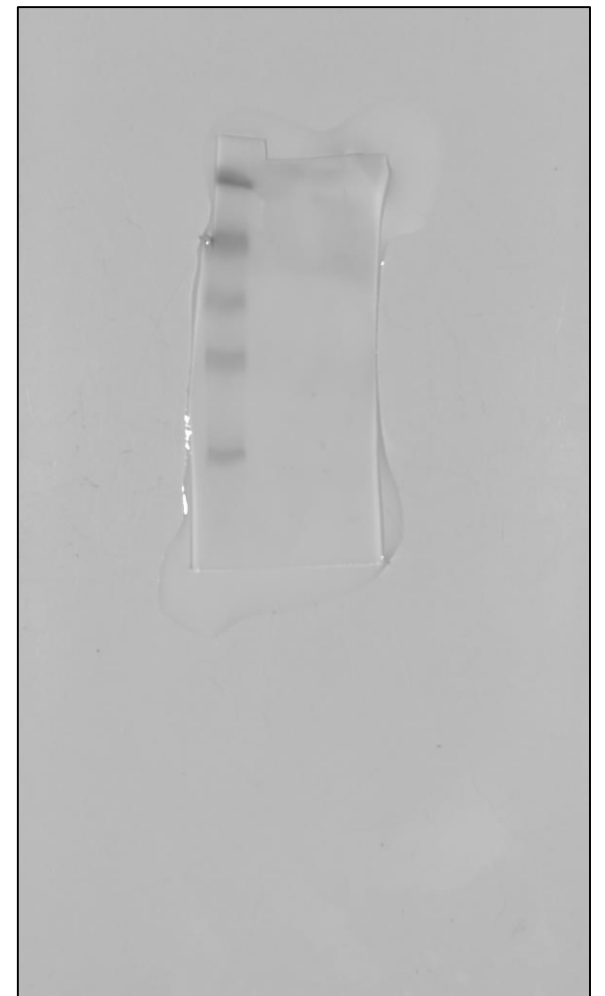

Vazyme (MP201)

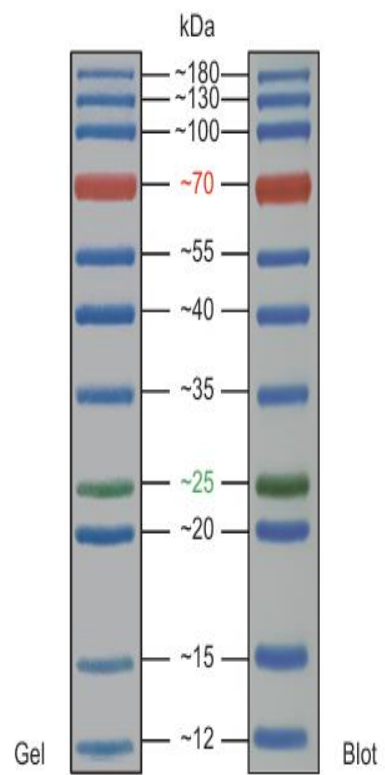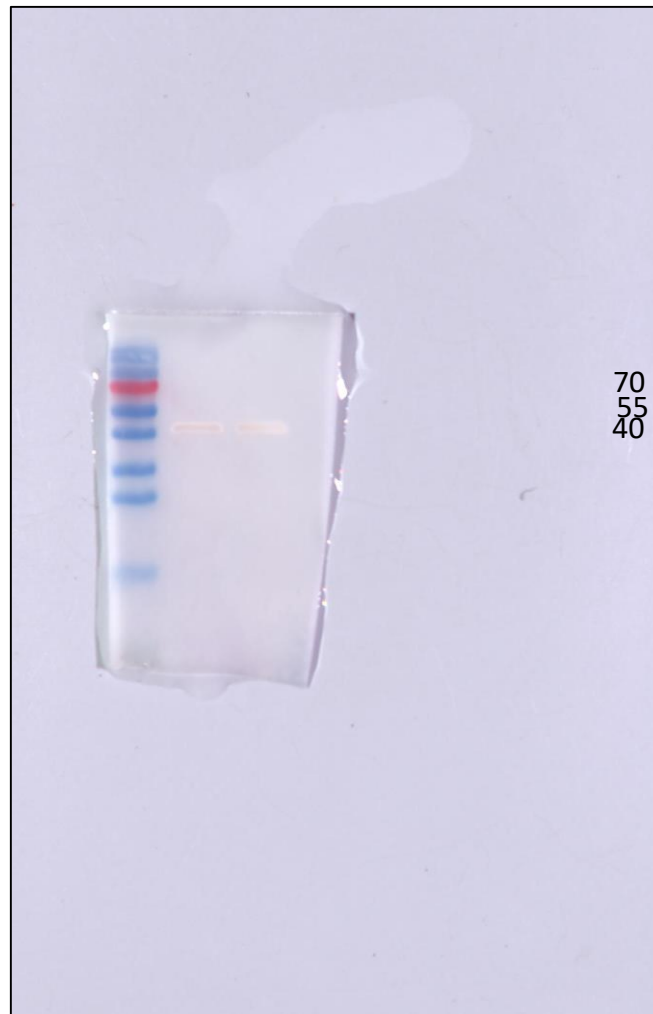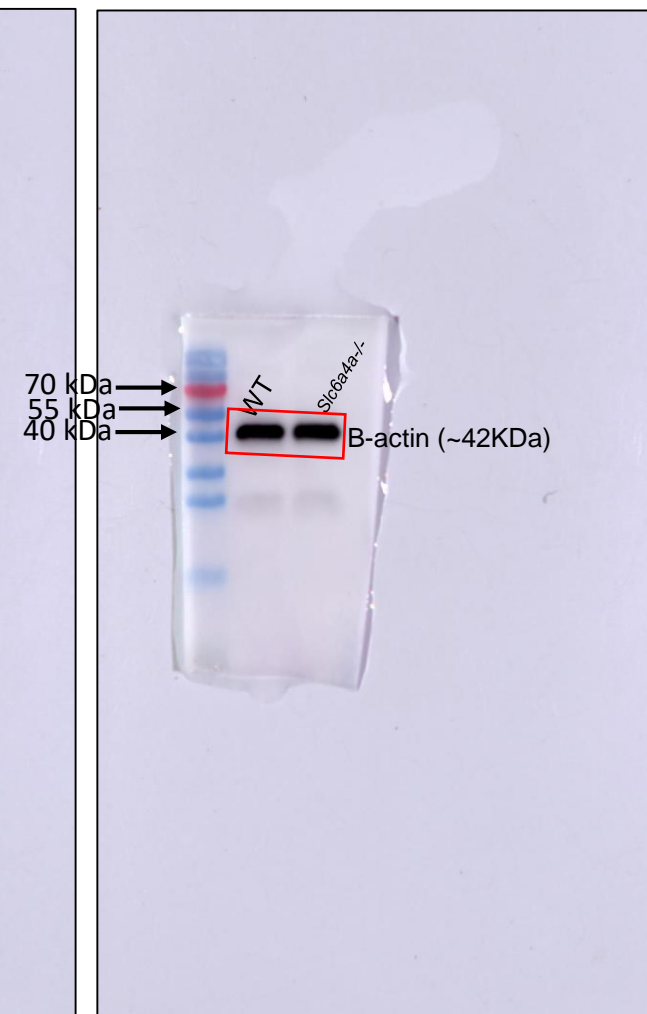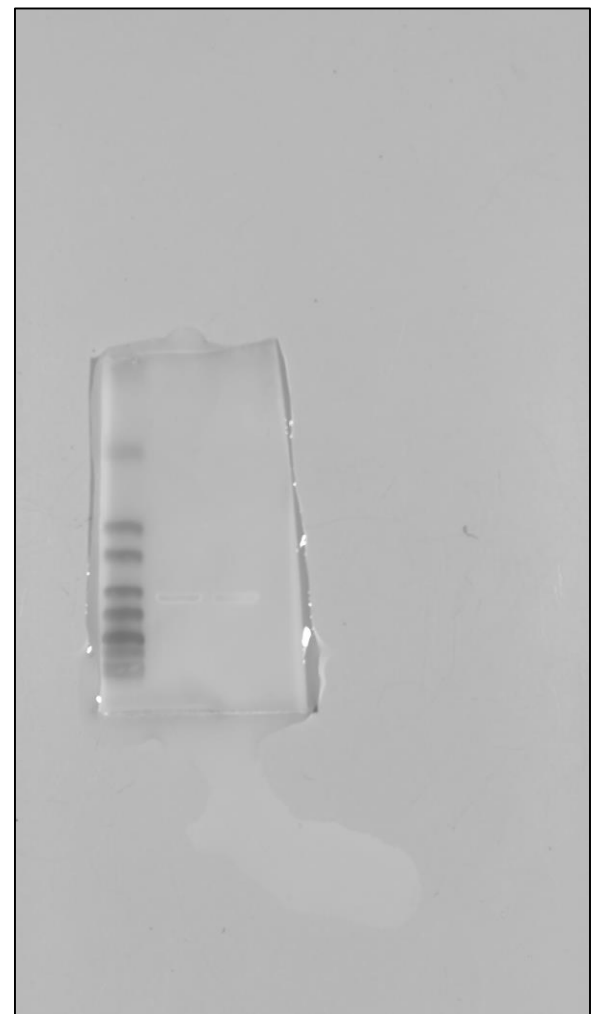

Fig 1. 12% Tris-glycine SDS-PAGE

Figure. 3.(C)

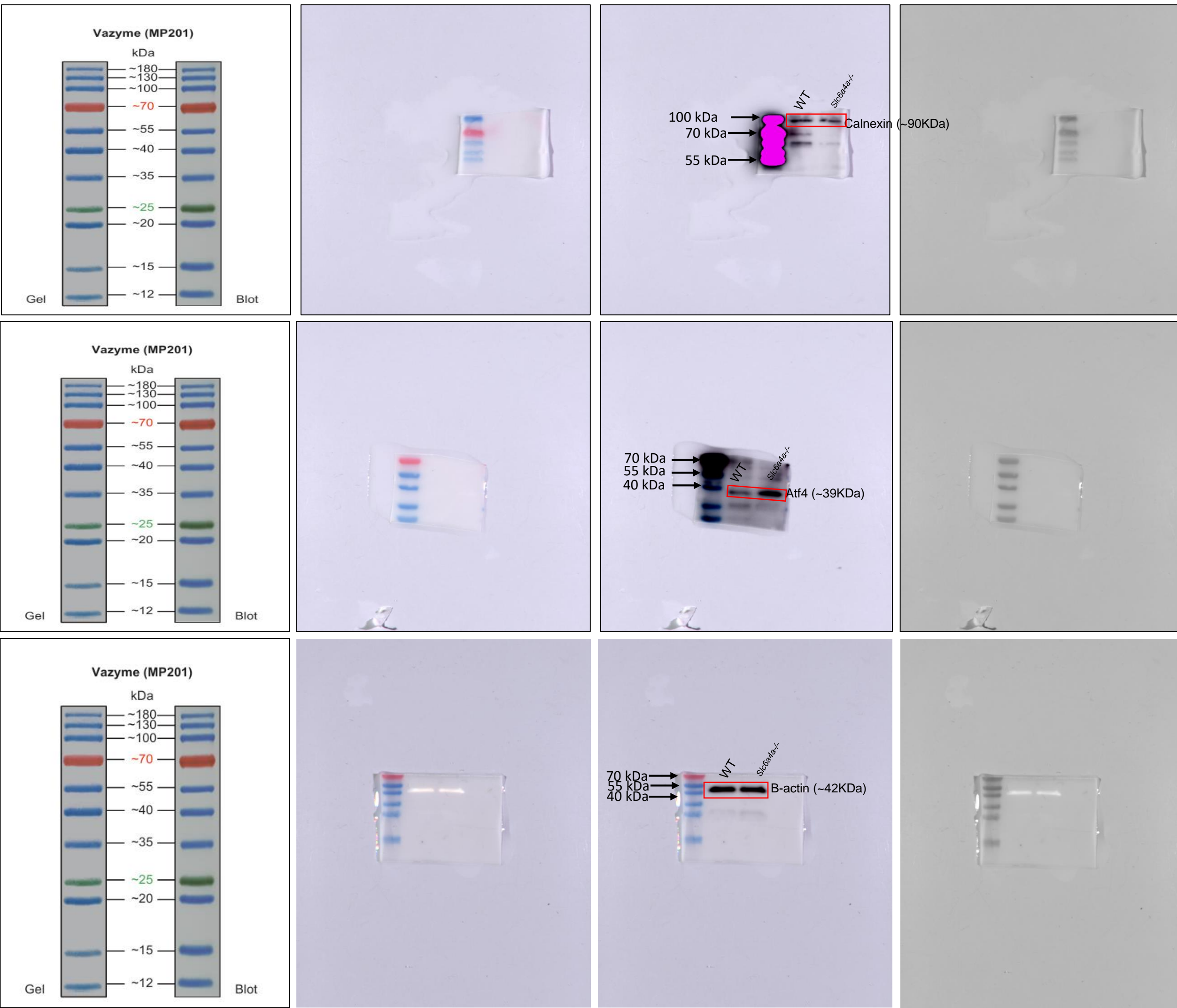

Fig.5(C)

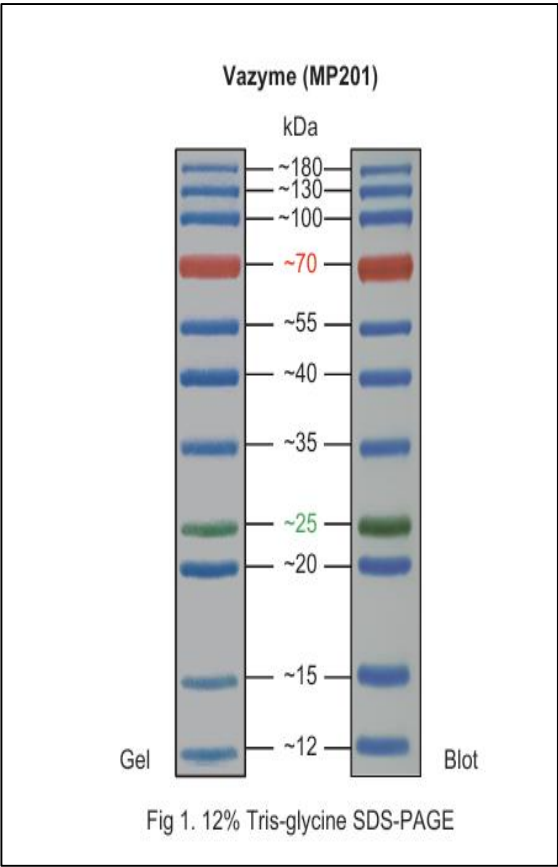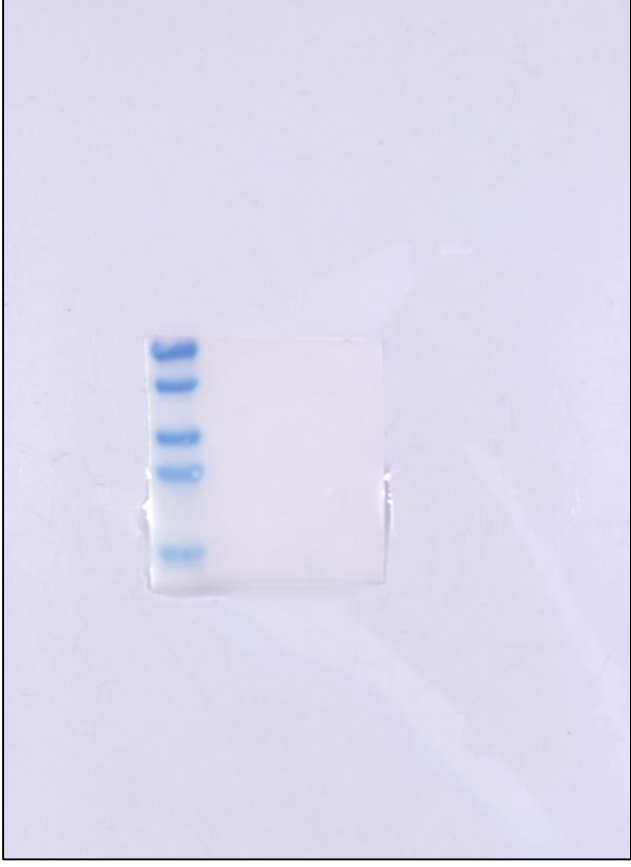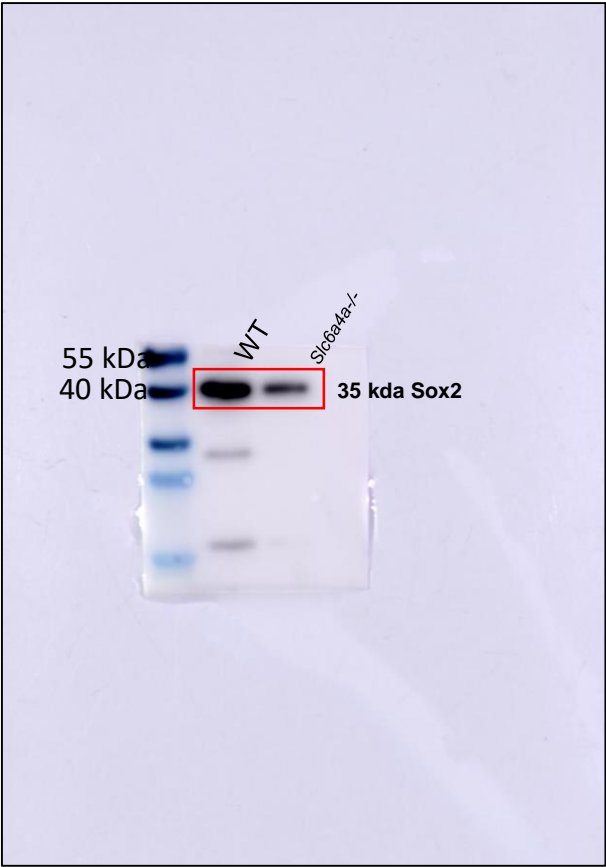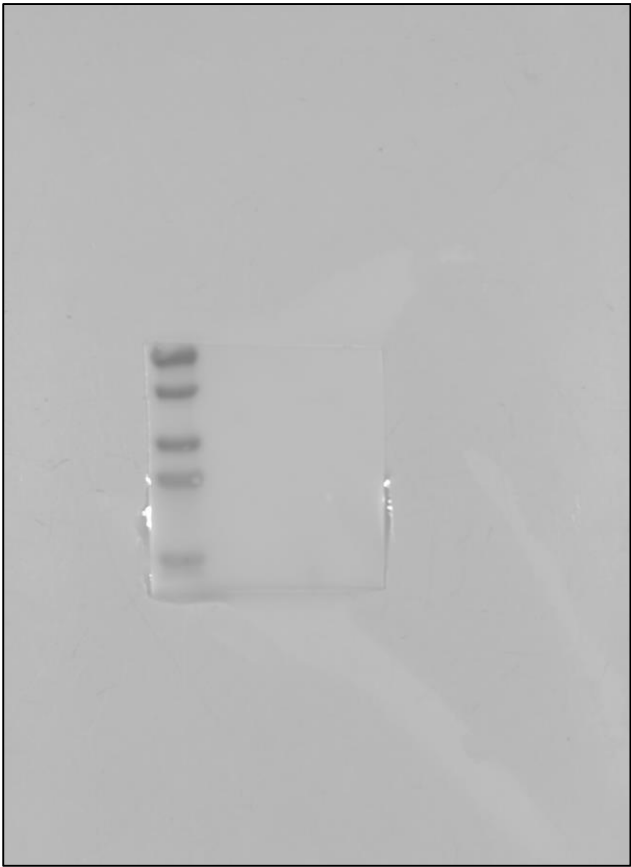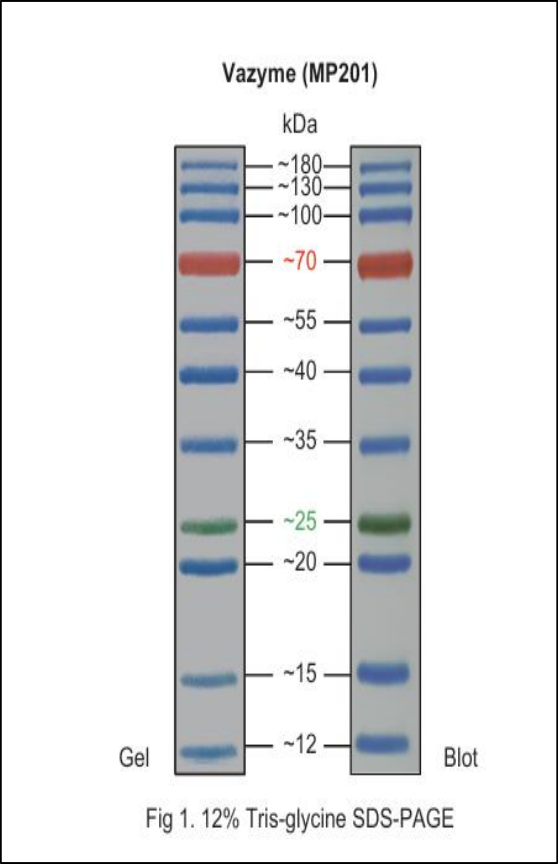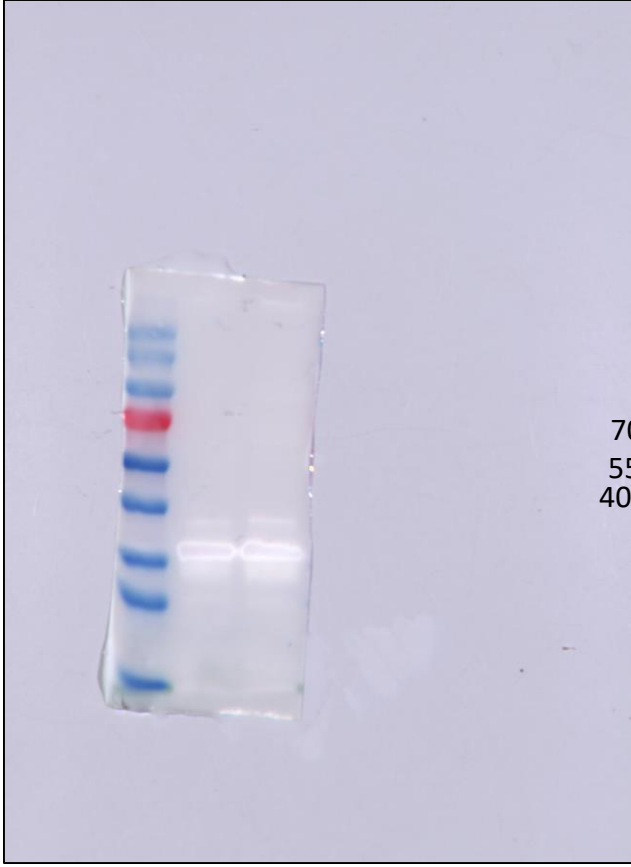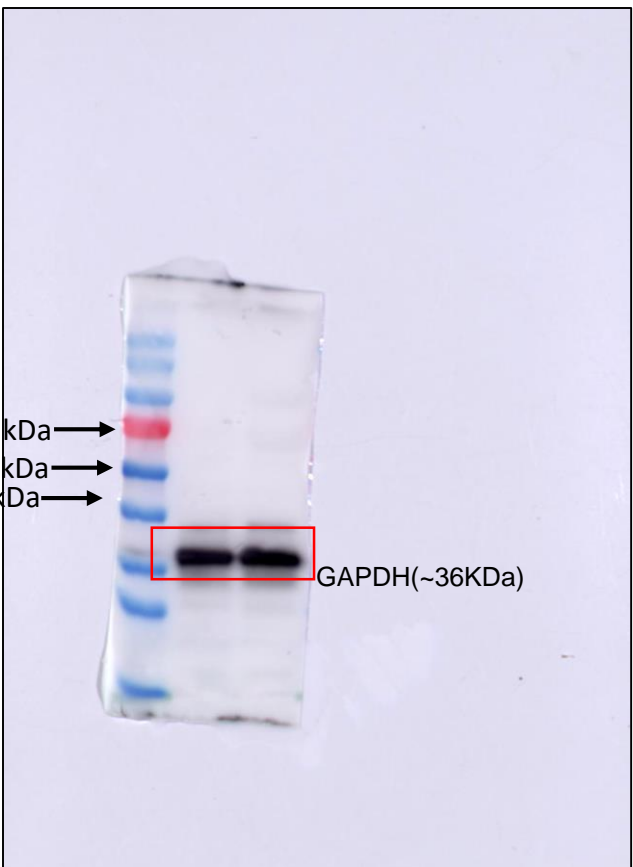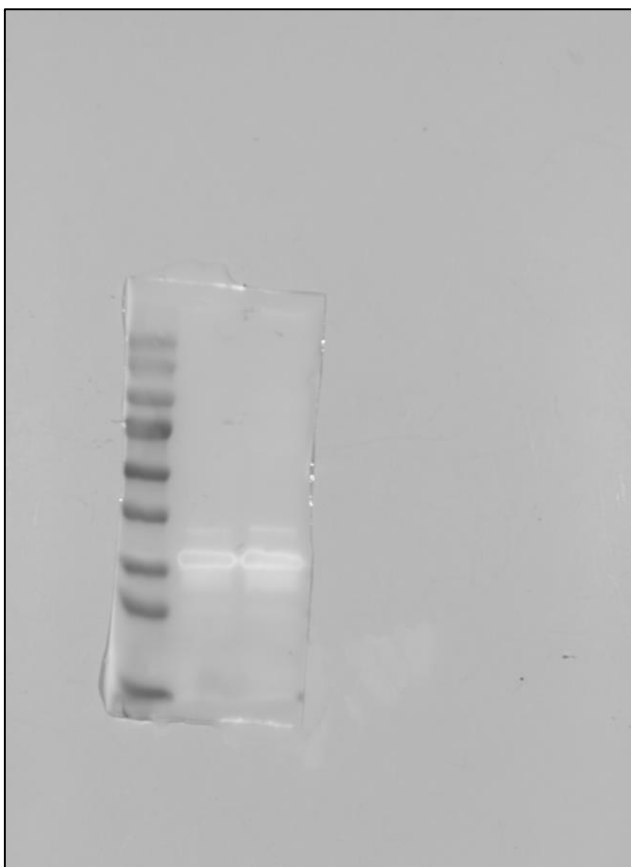

**Figure. 6(A)**

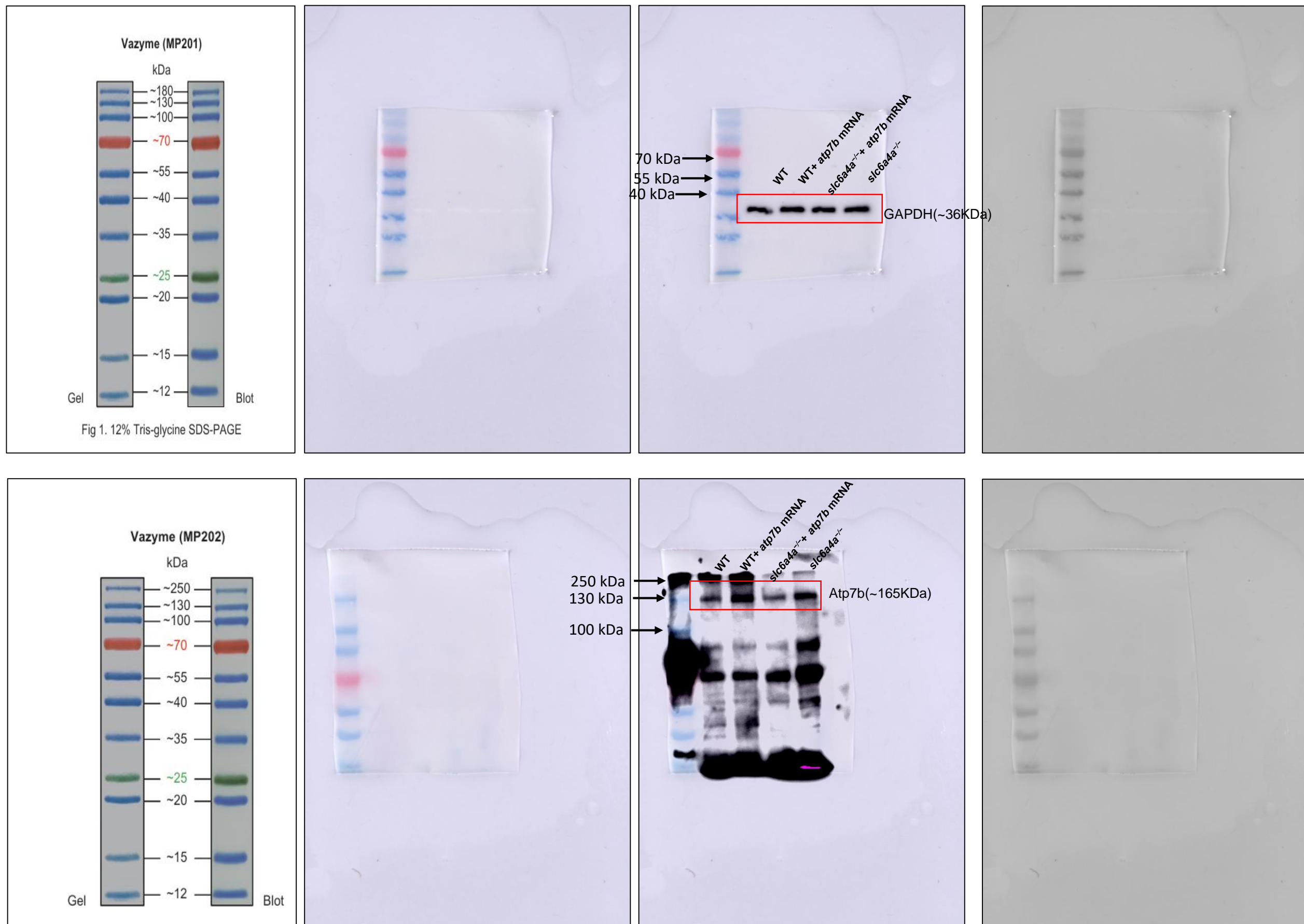

Figure. 8(B)

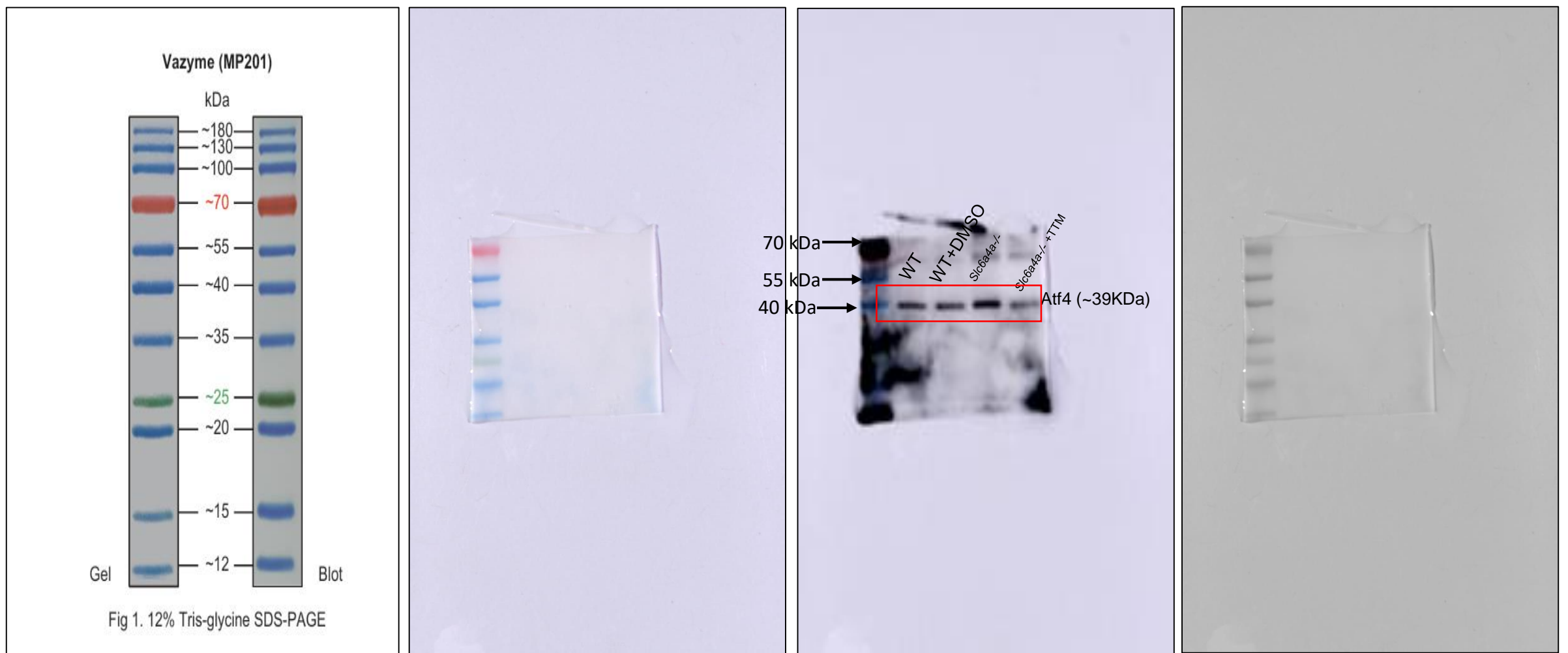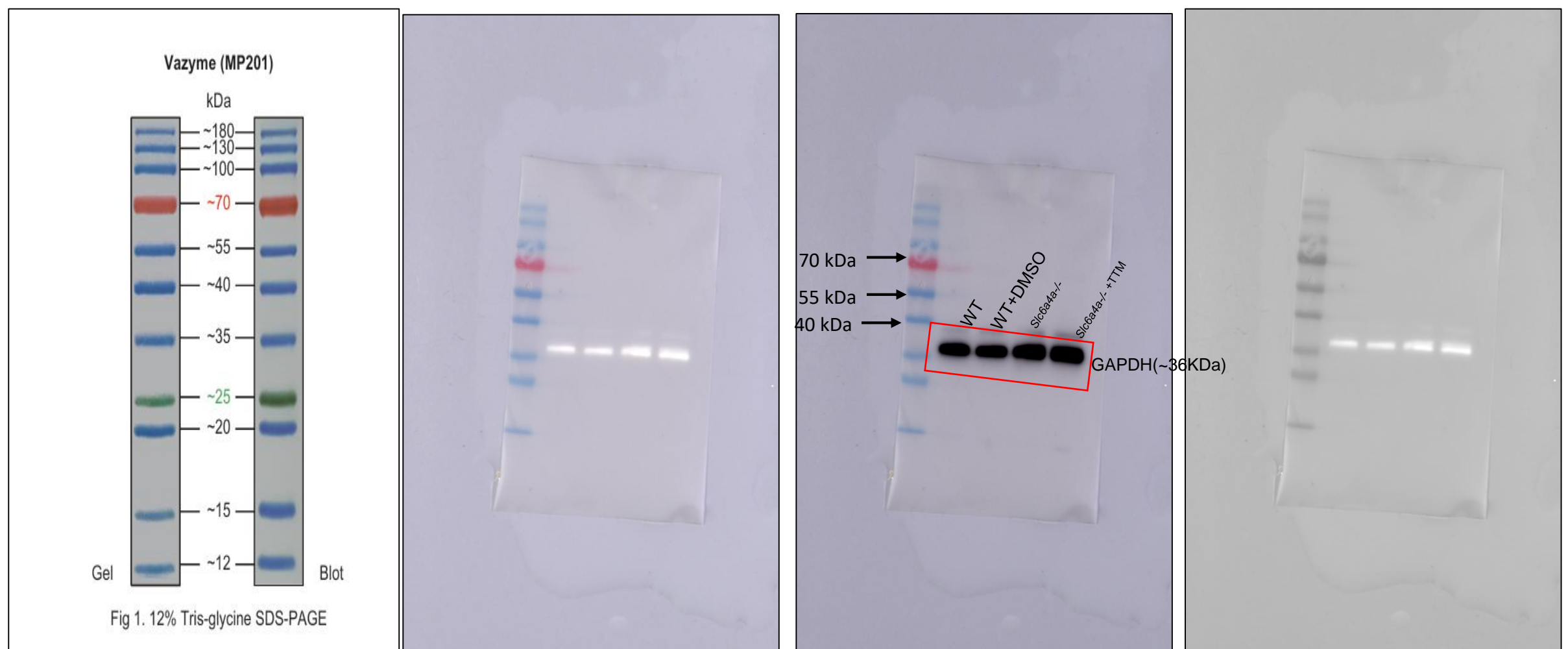

Supplement: Supplementary file 1 [file animals-16-02036-s001.zip › File S1. Western blotting original picture.pdf]
